# Statistics of dislocation avalanches in FCC and BCC metals: dislocation mechanisms and mean swept distances across microsample sizes and temperatures

**DOI:** 10.1038/s41598-020-75934-5

**Published:** 2020-11-04

**Authors:** Jorge Alcalá, Jan Očenášek, Javier Varillas, Jaafar A. El-Awady, Jeffrey M. Wheeler, Johann Michler

**Affiliations:** 1 Department of Materials Science and Metallurgical Engineering, InSup, ETSEIB, Universitat Politècnica de Catalunya, 08028 Barcelona, Spain; 2 New Technologies Research Centre, University of West Bohemia in Pilsen, 30614 Plzeň, Czech Republic; 3 Department of Mechanical Engineering, Whiting School of Engineering, The Johns Hopkins University, Baltimore, MD 21218 USA; 4 Laboratory for Nanometallurgy, Department of Materials, Swiss Federal Institute of Technology in Zürich, Vladimir-Prelog-Weg 5, CH-8093 Zürich, Switzerland; 5 Laboratory for Mechanics of Materials and Nanostructures, Empa, Swiss Federal Laboratories for Materials Science and Technology, 3602 Thun, Switzerland; 6 Present Address: Institute of Thermomechanics, Czech Academy of Sciences, 182 00 Prague 8, Czech Republic

**Keywords:** Nanoscale materials, Structural materials, Techniques and instrumentation, Theory and computation

## Abstract

Plastic deformation in crystalline materials consists of an ensemble of collective dislocation glide processes, which lead to strain burst emissions in micro-scale samples. To unravel the combined role of crystalline structure, sample size and temperature on these processes, we performed a comprehensive set of strict displacement-controlled micropillar compression experiments in conjunction with large-scale molecular dynamics and physics-based discrete dislocation dynamics simulations. The results indicate that plastic strain bursts consist of numerous individual dislocation glide events, which span over minuscule time intervals. The size distributions of these events exhibit a gradual transition from an incipient power-law slip regime (spanning $\approx$ 2.5 decades of slip sizes) to a large avalanche domain (spanning $\approx$ 4 decades of emission probability) at a cut-off slip magnitude ${s}_{\mathrm{c}}$. This cut-off slip provides a statistical measure to the characteristic mean dislocation swept distance, which allows for the scaling of the avalanche distributions *vis-à-vis* the archetypal dislocation mechanisms in face-centered cubic (FCC) and body-centered cubic (BCC) metals. Our statistical findings provide a new pathway to characterizing metal plasticity and towards comprehension of the sample size effects that limit the mechanical reliability in small-scale structures.

## Introduction

Crystal plasticity is characterized by the onset of intermittent dislocation glide events or avalanches, which lead to prominent strain burst emissions when the sample size is reduced to a few micrometers. The emission probability, $P$, of a dislocation burst of size $s$ is ascribed to the power-law relation $P\left(s\right)\propto {s}^{-\lambda }$, where the exponent, $\lambda$, is a universal parameter with scale-invariant value $\approx$ 1.5^1–11^. This arguably led to the interpretation in that the size distribution of the dislocation bursts fulfils a mean-field depinning phase transition paradigm characterized by a critical $\lambda$ exponent^3,7–10,12–14^. Truncation of intermittent responses is a key feature in the scaling of physical phenomena ranging from earthquakes to neural activity^15^. In crystal plasticity, $P\left(s\right)$ is exponentially truncated when the strain bursts approach a cut-off value. The notion of stress-tuned criticality (STC) then arises when this cut-off slip is governed by the applied stress, while volume-tuned criticality (VTC) implies that the cut-off depends on sample size. In both models, tuned criticality may arise at critical values of stress and sample volume^3,7,8,12,13^. Alternatively, in the advent of self-organization processes of the dislocation structure during straining, dislocation glide may occur at a fixed criticality level. The slip distribution then becomes independent of the applied stress, leading to the hypothesis of self-organized critically (SOC)^12,15–18^. Along these lines, it may be argued that the self-similar scaling of the dislocation cells arising at large shear strains during single crystal deformation may tentatively result in SOC.

The full-fledged evolutionary character of the dislocation network becomes manifest during plastic straining of macroscopic material samples, where the mobile dislocations are intermittently pinned by immobile forest counterparts through junction forming processes^18^. While the applied stress and dislocation density both fluctuate during subsequent avalanche emissions, a net increase in dislocation density prevails due to the storage of the mobile dislocations within the steady network, yielding a net positive slope $\theta$ in the stress–strain curve that quantifies onset of strain hardening^19^. This conception is markedly affected by the sample size. In the smaller micrometer and submicrometer-sized free-standing crystals, plasticity is governed by the scarcity of dislocation sources and by the onset of a single-ended dislocation source arrangement pinned at the sample bulk that intermittently produces dislocation avalanches. The term *confined plasticity* is then coined to embrace the notion in that the formation of the dislocation network is drastically affected by the limited sample size. Under these conditions, surface annihilation of mobile dislocations becomes a fundamental mechanism that prevents dislocation network development^20–27^.

A transition to bulk-like plasticity may occur in micrometer-sized samples where the incipient dislocation structure evolves into a heavily entangled (dense) network containing a myriad of dislocation sources that activate at large applied stresses. Dislocation multiplication thus outweighs surface annihilation. Bulk-like plasticity also develops in larger microcrystals with mildly entangled networks, where the interaction of the mobile dislocations with the forest arrangement already hinders significant surface annihilation. Since the dislocation density may approach similar values as in macroscopic scales, plastic straining occurs at similar levels of applied stresses^2,18,19,28–30^.

Computational simulations have shown that the interaction between mobile and forest dislocations affects the avalanche distributions. The seminal mean-field understating to this result is that with the increasing dislocation entanglement that occurs upon the onset of subsequent strain bursts, the distance to the critical driving stress remains constant which results in the attainment of an intermittent, steady-state response in the context of SOC. The cut-off slip size then becomes independent of the applied stress^4,7,12,16–18^. Tentatively, STC would then prevail under vanishing hardening ($\theta$ $\to 0$), where greater cut-off slip magnitudes result with increasing applied stresses. Recent investigations have however challenged the above mean-field depinning scenario^13,14^, showing that the avalanche distribution crosses over between two power-law avalanche regimes at a critical cutoff slip size^14^. Experimental evidence is still needed in support of these conceptions, including the influence of the characteristic face-centered cubic (FCC) and body-centered cubic (BCC) dislocation glide mechanisms in micro-scale crystals deforming under confined and bulk-like plasticity.

Here, we investigate the dislocation mechanisms and statistical features of the individual dislocation glide events which were concealed in the previous investigations of strain bursts in micro-scale sample sizes. A comprehensive set of strict displacement-controlled microcrystal compression experiments and physics-based simulations was performed to gain access to these individual intermittent events, spanning over few nanoseconds, which are regarded as the fundamental constituent elements to dislocation-mediated plasticity. We show that the size distribution of the individual dislocation glide events is characterized by a transition from an incipient power-law slip regime to a large avalanche domain. This transition is a fingerprint of the dislocation glide phenomenology developing in FCC and BCC microcrystals, which governs the onset of confined and bulk-like plasticity in micrometer-sized samples along with the competition between STC and SOC.

## Methods

### Micropillar compression experiments

Our microcrystal compression experiments, performed under strict displacement control, enabled the identification of individual dislocation glide events. These are the constituent plastic intermittencies which occur within the strain bursts observed in experiments driven with conventional dynamically-reactive testing systems (see Supplementary Material, Section 1, and the current Results and discussion section for a description on the influence of the straining mode and testing system on the plastic intermittencies). The cylindrical pillar-like microcrystals used in our compression experiments were focused ion beam (FIB) milled from [111]-oriented Cu and Ta single crystals, a [100]-oriented Al single crystal, and a [100]-oriented W single crystal. Pillar-like rectangular microcrystals with square cross-sections were also FIB-milled from a [100]-oriented Cu single crystal and a [123]-oriented Al surface. Beam current in the Ga source was reduced to less than 200 pA in all final machining steps to reduce damage. The cylindrical Al microcrystals had a diameter $D\approx$ 8 µm whereas the Cu, Ta and W counterparts had $D$ ranging from 0.5 to 20 µm. The effective diameters of the rectangular Cu microcrystals ranged from $D\approx$ 0.5 to 4 µm. In all cases, the sample height, $l$, was $\approx$ 3$D$. The rectangular samples were heat treated at 600 °C for 1 h to remove any FIB-induced dislocation loops^31^.

The compression experiments were performed using a modified Alemnis nanoindenter (Alemnis GmbH, Thun, Switzerland) fitted inside the high vacuum chamber of a Zeiss DMS 962 scanning electron microscope (SEM). This system is capable of operating under strict, intrinsic displacement control, thus maintaining indenter tip position during sudden avalanche emissions (Section 1 in Supplementary Material). The nanoindenter was custom modified to enable testing at high temperatures with the sample and indenter temperature matched to minimize any influence of thermal expansion drift on applied displacements^32^. The applied strain rates, $\dot{\varepsilon }$, ranged from ${10}^{-4}$ to ${10}^{-3}$, which is sufficiently small so as to neglect additional straining produced during avalanche propagation (Section 2 in Supplementary Material). A detailed account of some of the stress–strain curves is published elsewhere^18,31,33^.

### MD and DDD simulations

Molecular dynamics (MD) simulations were conducted by uniaxially compressing cuboidal [110]-oriented Al and cuboidal [100]-oriented Ta simulation cells with periodic *and* non-periodic boundary conditions; where the dislocation density, $\rho$, ranged from $\approx {10}^{15}$ to ${10}^{16}$ ${\mathrm{m}}^{-2}$ to mimic bulk-like plasticity at extreme dislocation densities. The simulation cell size was 70 $\times$ 70 $\times$ 40 nm comprising $\approx$ 11 million atoms for Al and $\approx$ 10 million atoms for Ta, modelled through the embedded-atom method (EAM) potentials in Refs.^34,35^. All simulations were carried out with the LAMMPS code^36^ under an NPT ensemble, where the uniaxial stress $\sigma$ is obtained from the pressure tensor in the direction of the applied displacement (with a frequency of 200 fs) while the barostat acts in the other main directions of the simulation cell. The Al cells were deformed at RT and the Ta counterparts at RT and 630 °C with $\dot{\varepsilon }$ of 1.5 $\times {10}^{6} {\mathrm{s}}^{-1}$. The networks were introduced into the computational cells by bringing one of the free surfaces into contact against a spherical indenter until a dense defect network attained (see^37^ and Sects. 6 and 7 in the Supplementary Material). Upon application of periodic boundary conditions to the simulation cells, the stress–strain curves were taken to represent bulk material responses. The freestanding MD simulations were then exclusively employed to assess slip trace formation at the surface. Details of the strengthening provided by the incepted networks (see Ref.^38^) are given in Sect. 6 of the Supplementary Material.

The 3D DDD simulations were conducted with an in-house version of the open source code ParaDis^30,39^ to capture confined plasticity responses where dislocation annihilation occurs at the free surfaces. All dislocation reactions are planar in this in-house version of the open source code, further incorporating a set of atomistically-informed, physics-based cross-slip mechanisms, the details of which are described in^30^. The assumed elastic material properties matched those of FCC Ni (Young's modulus *E* $=$ 210 GPa, and Poisson's ratio *ν* = 0.31). The stress–strain curves were obtained by uniaxially compressing rectangular computational cells with a square cross-section under a nominal strain rate control of $\dot{\varepsilon }=$ 200 s^−1^ (where the stress $\sigma$ along the straining direction is evaluated with a frequency of 5 ns). Free-standing [100]-oriented microcrystals were compressed with $D=$ 2 and 5 μm with aspect ratio $l/D=3$. The initial dislocation microstructure was introduced as Frank-Reed (FR) sources having random line directions randomly assigned to one of the twelve FCC slip systems. The dislocation density, $\rho$, was varied in the range of ${10}^{14}$ m^−2^ to reproduce the attainment of bulk-like plasticity and the slip distributions from the experiments with Cu microcrystals, while the applied $\dot{\varepsilon }$ remained fixed at 2$\times {10}^{2}$ s^−1^. DDD simulations under severe dislocation starvation were also performed in smaller microcrystals with $D=$ 1 μm ($\rho =$ ${10}^{12}$ m^−2^). Since the long-range image field resulting from the traction-free boundary conditions give rise to relatively small corrections in the current simulation cells^40,41^, this image field is ignored here.

### Measurement of the slip distributions

**Figure 1:**
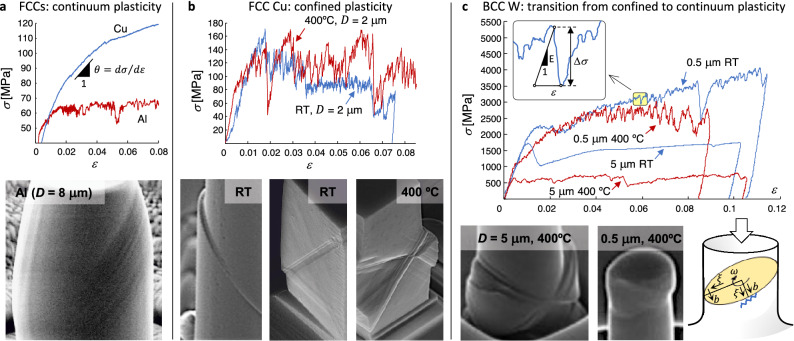
Stress–strain curves and slip phenomenology in FCC and BCC microcrystals. (**a**) Continuum (bulk-like) plasticity characterized by constant shear strain *γ* along the sample height and by a homogeneous slip trace distribution. Note the onset of marked strain-hardening for Cu (*D* ≈ 20 μm, large θ) and mild strain-hardening for Al (*θ* → 0). (**b**) Confined plasticity characterized by markedly serrated, heterogeneous slip traces along the {111} <0$\overline{1}$1> slip system family^31^. Notice the development of stress drops in the stress–strain curves, a feature that is associated with recurrent single-ended dislocation source operation and the occurrence of sudden dislocation network destabilization processes, which lead to large values of the Pearson correlation coefficient, *r* (see Fig. 5). (**c**) BCC plasticity across sample sizes^33^. Increasingly fluctuating stress–strain curves, characterized by the largest *r* values, are observed with decreasing micropillar diameter and increasing temperature. The inset illustrates the emission of an individual slip event under progressive straining (with the current, intrinsic displacement controlled testing system) characterized by Young’s modulus, *E*, stress drop, Δ*σ*, and plastic strain increment, *ε*. The schematic representation of a cylindrical micropillar illustrates the alternating screw/edge dislocation characters of an active singleended source revolving at the micropillar center. This leads to the onset of wavy slip as shown for *D* = 0.5 μm at 400 °C. Heterogeneous slip trace distributions along the <111> {11$\overline{2}$} slip system family is also illustrated for *D* = 5 μm at 400 °C.

Representative stress–strain curves under true displacement control in conjunction with the associated surface slip traces are shown in Fig. 1. In our experiments and simulations, an individual slip event involves sudden instability of the dislocation arrangement producing stress drop, $\Delta \sigma$, at fixed displacement (see inset to Fig. 1c). The onset of a slip magnitude, $s$, thus reduces the sample height, $l$, so as to induce stress drop, $\Delta \sigma$, as the elastic energy is released^37,42^. This process is described through

1 $$s=\left[l\Delta \sigma /(E \times SF)\right],$$

where $SF$ is the Schmid factor and $E$ is the effective elastic modulus of the microcrystal (see Sect. 2 in Supplementary Material). Equation (1) indicates that the stress drop magnitude $\Delta \sigma$ increases when the same slip event size occurs in samples of a smaller height. This supports the notion in that the plastic intermittencies may only become noticeable in small (microcrystalline) sample sizes. According to coarse-grained crystal plasticity^43^, the following correlation between the slip magnitudes and the shear strain $\gamma$ can then be expressed

2 $$s=\gamma l=\left(Nb{A}_{\mathrm{s}}/V\right)l=\frac{NbL}{D},$$

where $b$ is the magnitude of the Burgers vector, $N$ is the total number of mobilized dislocations in a given avalanche, $L$ is the mean distance swept by each dislocation, ${A}_{\mathrm{s}}=LD$ is the swept area, and $V$ is the sample volume. Equation (2) prescribes the possible onset of fractional slips ($s<1b$) when a small number of mobile dislocations glide across a distance $L\ll D$. In all coarse-grained analysis based upon Eq. (2), the parameter $L$ becomes a linear representation of the *area* swept by the expansion of the unzipped three-dimensional dislocation segments along with the revolving of the surface truncated single-ended dislocation sources in the active slip plane.

The avalanche size distribution was investigated by recourse to the complementary cumulative distribution function, $C(s)$, which gives the emission probability for a slip event $s\ge {s}_{\mathrm{min}}$ (where ${s}_{\mathrm{min}}$ is the minimum measurable slip). Hence, $C\left(s\right)\to 1$ when $s\to$ ${s}_{\mathrm{min}}$. For a power-law probability density function $P(s)\propto {s}^{-\lambda }$, where $C\left(s\right)\equiv {\int }_{s}^{\infty }P\left(s\right)\mathrm{d}s$, it follows that $C\left(s\right)\propto {s}^{-\kappa }$ where $\kappa =\lambda -1$. Further analyses in the time domain were performed through the currently defined avalanche emission frequency distribution function $\nu (s)=n(s)/{t}_{\mathrm{T}}$, where $n(s)$ is the number of avalanches that carries slip greater than $s$ and ${t}_{\mathrm{T}}$ is the total straining time. A detailed description of these functions and their properties are given in the Supplementary Material.

## Results and discussion

### Individual avalanche emissions versus dynamically-driven bursts

An important finding from the current MD and DDD simulations is that microscale plasticity is essentially characterized by the onset of individual avalanche events associated with recurrent stress drops $\Delta \sigma$. Our simulations show that these individual avalanches are emitted regardless of the externally applied deformation rate, which may become several decades greater than those in conventional dynamically-driven experiments. In these experiments, the sample is rapidly strained after the onset a plastic instability, the magnitude of which is then controlled by the activation the feedback loop and mechanical dynamics of the testing system (see the discussion given in Sect. 1 of the Supplementary Material).

Since the MD simulations indicate that the propagation time frame is $\approx$ 20 ns for a medium-sized avalanche event sweeping across a microcrystal with $D=$ 2 µm, it is argued that a larger, dynamically-driven, strain burst ($\Delta \varepsilon \approx$ 0.1) occurring over a time frame several decades greater would be comprised of more than a hundred of individual avalanches. Within the range of time frames under consideration, each constituent individual avalanche would essentially propagate at fixed displacement, as reproduced with the current experiments, which results in the $C\left(s\right)$ distributions being much narrower than those of the dynamically-driven strain bursts. A correspondence is therefore anticipated between the $C\left(s\right)$ distributions from experiments and simulations performed under strict displacement control and those of the constituent individual avalanche events concealed in the dynamically-driven experiments. Much smaller $\nu (s)$ are however reached in the current displacement controlled experiments, where low strain-rates of $\dot{\varepsilon }= {10}^{-3}$ to ${10}^{-4} {s}^{-1}$ are externally applied, than in the computational simulations or in the dynamically-driven strain bursts where $\dot{\varepsilon }$ is several decades greater.

Finally, the MD simulations illustrate the energetics of the current individual slip events. It is found that within the large avalanche domain, the elastic energy *release* rate is 20 times greater than the elastic energy *storage* rate prior to the onset of the intermittency. The ratio between these elastic energy rates is reduced to 4 within the small slip domain, while it approaches unity for $s<0.1b$ where plastic deformation proceeds through smooth dislocation bowing processes ($\Delta \sigma \to 0$).

### Universal scaling of slip distributions

**Figure 2:**
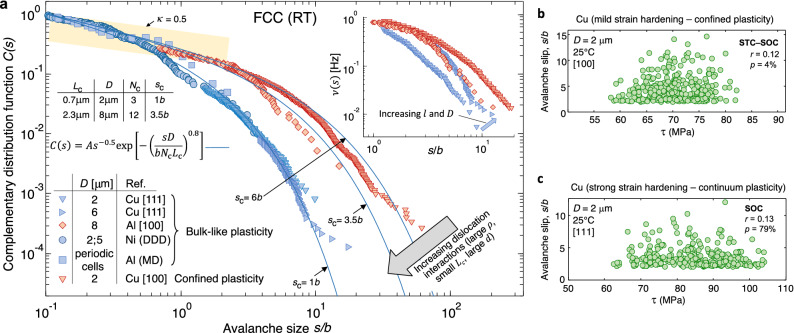
(**a**) Master distributions of the individual slip events in FCC microcrystals from experiments and simulations under bulk-like and confined plasticity. In accordance with the universal *C*(*s*) function included in the main figure (Eq. (4) in the main text), notice the onset of an incipient slip domain, characterized by power law exponent *κ* ≈ 0.5, and an exponentially-truncated large avalanche domain which sets-in beyond the cut-off slip size *s *> *s*_c_ (where the band with power-law exponent *κ* = 0.5 is drawn for visual guidance). The agreement between the *C*(*s*) distributions from experiments, MD and DDD simulations of microcrystals deforming under bulk-like plasticity is further shown, where the parameters *L*_c_ and *N*_c_ in the Table provide the scaling of the *C*(*s*) distributions as function of sample diameter *D*. The inset to (**a**) provides avalanche emission frequency function ν(*s*) from key experiments with the same color code as in the main figure. Scattered slip events are then given in (**b**) and (**c**), where the possible correlation between the slip sizes and the applied shear stress, *τ*, suggests the onset of stress-tuned criticality while the lack of correlation supports self-organized criticality (SOC), as discussed in the main text.

**Figure 3:**
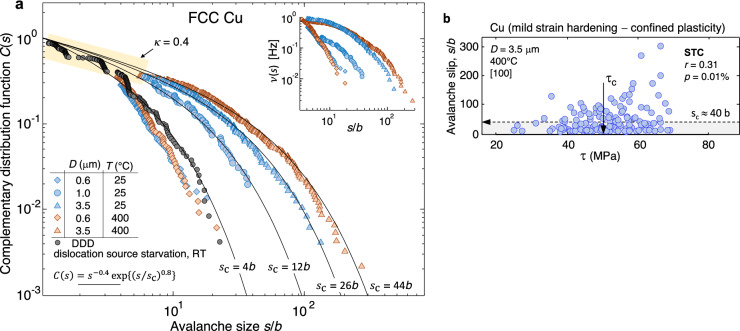
Extension of the slip distributions in Fig. 2 using a different set of [100]-oriented Cu microcrystals tested as a function of temperature and size. These microcrystals were heat treated to remove the FIB-induced dislocations, and exhibited confined plasticity when subjected to uniaxial compression (see Fig. 1b). As compared to in the Cu micropillars in Fig. 2, the current incipient slip regime (*κ* ≈ 0.4) is clearly prolonged to larger cut-off slip sizes in the range of *s*_c_ ≈ 10*b*–20*b*. This feature becomes more evident as the testing temperature is increased (*D* = 3.5 μm at *T* = 400 °C). Notice that when the microcrystal diameter is reduced towards sub-micrometer sizes, the cut-off slip *s*_c_ is drastically reduced. This is supported by the DDD simulations performed with a reduced number of dislocation segments to capture such source starvation responses (grey circles, where *D* = 1 μm, *ρ* = 1 × 10^12^ m^−2^ and *N* = 1 along with the mechanisms illustrated in Fig. 4e,f). The inset provides avalanche emission frequency function *C*(*s*) from the experiments in the main figure. The scattering of the slip events as a function of the applied shear stress *τ* in the active slip systems in shown in (b), in support of stress-tuned criticality (STC). Larger slip events thus occur when *τ* > *τ*_c_.

Master slip distributions as a function of microcrystal orientation, size and crystalline structure are shown in Figs. 2, 3 and 5. These figures incorporate the individual slip events from our strict displacement controlled experiments, MD and DDD simulations. Overall, the slip distributions comply with

3 $$C\left(s\right)=A{s}^{-\kappa }{\mathrm {exp}}\left[-{\left(\frac{s}{{s}_{\mathrm{c}}}\right)}^{n}\right],$$

where the universal power-law exponent $\kappa \approx$ 0.4–0.5 $\pm 0.03$ ($\lambda \approx$ 1.4–1.5) describes the incipient slip regime attaining for $s<{s}_{\mathrm{c}}$, $n$ approaches 1.0, and $A$ is a normalization constant which ensures $\underset{0}{\overset{\infty }{\int }}P\left(s\right)\mathrm{d}s\equiv 1$, so that by virtue of the scaling properties of function $C\left(s\right)$ in Sect. 3 of the Supplementary Material, $A=1/{s}_{\mathrm{min}}^{-\kappa }$. The cut-off slip size ${s}_{\mathrm{c}}$ then sets the exponential truncation for ${s>s}_{\mathrm{c}}$, which results in the onset of a large avalanche regime with reduced emission probability. It is noted that while a similar formulation as in Eq. (3) was proposed in Ref.^4^, the distribution of the *individual* dislocation glide events is in better accord with the currently employed relation (see Sect. 5 in the Supplementary Material). Along these lines, the statistical scatter of the power-law exponent $\kappa$ in the small slip domain is computed here through the standard bootstrapping resampling method, where the hypothesis of the power-law scaling satisfies the Kolmogorov–Smirnov test. This involves comparison of the experimentally measured slip distributions against a synthetic set of 5000 power-laws with 5% significance level^44^, see Sect. 3 in the Supplementary Material.

The background to the scaling of the slip distributions stems from Eq. (2), where it follows that ${s}_{\mathrm{c}}=b{(NL)}_{\mathrm{c}}/D$. The substitution of this relation into Eq. (3) yields the explicit dependency of the slip distributions upon the microsample diameter, $D$, and the total distance travelled by the mobile dislocations in an intermittency with $s={s}_{\mathrm{c}}$, as given by the product ${(NL)}_{\mathrm{c}}$. Along the seminal dislocation storage-recovery model^45^, detailed investigations in FCC crystals have revealed the intricate connection between a mean, deterministic value of the dislocation swept distance $L$ and the strain hardening response produced by the expansion of pinned dislocation segments across an arrangement of forest dislocations, leading to the formation of orientation-dependent junctions^19,46,47^. In the light of these investigations, we find $L=$ 0.7 μm and 2.3 μm for the Cu and Al microsamples with $D=$ 2 μm and 8 μm, respectively. This is consonant with the estimated $\rho \approx$ 7 $\times$ 10^14^ m^−2^ and $\rho \approx$ 8 $\times$ 10^13^ m^−2^ which develop under the applied shear stresses of ${\tau }_{\circ }\approx$ 65 MPa and 27 MPa in the microcrystal compression experiments of Cu and Al, respectively (see Sect. 6 in the Supplementary Material). It is noted that the strain hardening parameter $\theta$ is then reduced as compared to that attaining at smaller dislocation densities^18^.

### Mechanisms and statistics of bulk-like plasticity in FCC microcrystals

Figure 2 shows the $C(s)$ distributions of the individual slip events from experiments and simulations of bulk-like microscale plasticity, where a rather uniform arrangement of non-localized, fainting slip traces develops along the microcrystal height in conjunction with plastic barreling (Fig. 1a). A master slip distribution is constructed for microcrystals that exhibit intense interactions between the mobile dislocations and the forest dislocations. This distribution is fitted with ${s}_{\mathrm{c}}\approx$ 1$b$, and incorporates the experimental results from Cu microcrystals ($D\approx$ 2 μm) compressed along the [111] orientation, the DDD simulations for freestanding Ni microcrystals containing a large dislocation density, and the MD simulations for an extremely entangled Al bulk modeled through periodic boundary conditions.

**Figure 4:**
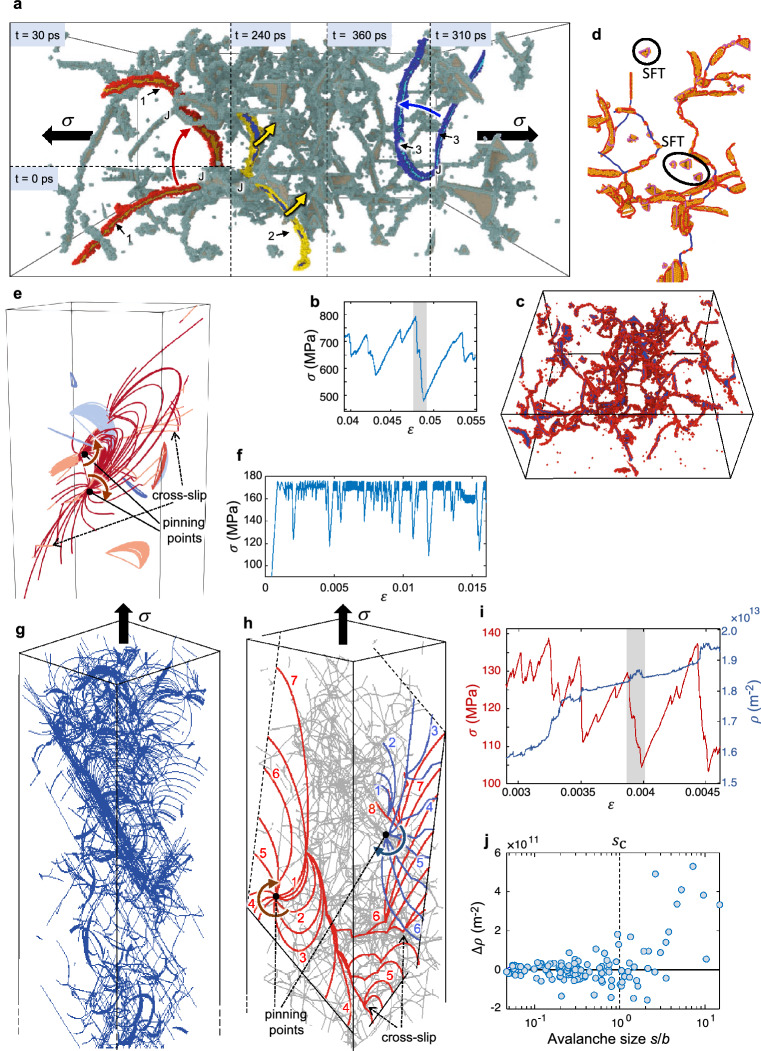
Mechanisms of the individual ultrafast slip events from the MD and DDD simulations in FCCs. (**a**–**d**) MD simulations for FCC Al deforming under bulk-like plasticity (see Supporting Movie S1). The slip event marked in grey in (**b**) underlies sudden dislocation expansions in (**a**), where segment 1 first revolves about junction J (*t* = 0–30 ps) while segment 2 subsequently glides towards the back of the computational cell (*t* = 240 ps). Segment 3 finally expands towards the front (*t* = 310–360 ps). The forest dislocation network is shown in (**c**) and (**d**), where glissile dislocations dissociate into leading and trailing partials, marked in red in (**d**). Sessile junction-type segments, highlighted in blue in (**d**), and isolated stacking fault tetraedra (SFT) are also observed. (**e**–**f**) DDD simulations of a Ni microcrystal that deforms under severe dislocation source starvation (*N* = 1). The composite plot in (**e**) overlies the evolution of the single mobile segment over time (in red) as it expands and revolves around the pinning points. The mobile segment eventually cross-slips into the intercepting systems while smaller, secondary dislocation segments (highlighted in different colors) bow under stress. The resulting stress − strain curve where plasticity attains under a constant stress level is shown in (**f**). (**g**–**j**) DDD simulations for Ni micropillars deforming under intense dislocation interactions (*s*_c_ ≈ 1*b*; *L*_c_ ≈ 0.5 μm). (**g**) shows stress drops and dislocation density evolutions within a small strain increment. A specific avalanche, marked in grey in (**i**), bifurcates into two active {111} < 1$\overline{1}$0> slip systems in (**e**). (**f**) illustrates that the avalanche initiated through Frank–Read source operation (1 − 3), producing a single-ended source (in red) which leads to slip trace formation (4–6) and the storage of dislocation segment (7) within the immobile forest. Expansion of segment (3), in red, then produces multiple cross-slip events and a surface slip trace (3 − 6). The segment is stored at (8), finally triggering activation of a secondary segment marked in blue (1 − 6). (**h**) shows the variation in dislocation density Δ*ρ* for the incipient slip (*s* < *s*_c_) and large avalanche domain (*s* > *s*_c_).

The dislocation mechanisms underlying the above master slip distribution are shown in Fig. 4. The MD simulations illustrate that the incipient slip domain is distinguished by correlated dislocation glide events involving the expansion of pinned loops over $L<30$ nm. These processes occur throughout the entire cell volume (Fig. 4a–d). In the MD simulations, stress drops $\Delta \sigma$ are no longer detected below the minimum slip size ${s}_{\mathrm{min}}$ $\approx 0.07b$ where the stress–strain curves acquire a continuous appearance and deformation is accumulated through reversible bowing of pinned dislocation segments along with the onset of gradual, smooth dislocation glide events over $L\approx 5$ nm. As the sudden release of elastic energy vanishes in these processes, they are not regarded as true avalanches.

While the above observations concern bulk-like responses, the DDD simulations incorporate the role of the free surfaces in avalanche propagation at dislocation density levels rendering similar $C(s)$ distributions as those measured experimentally with the Cu microcrystals. Overall, the DDD simulations indicate that when ${s\to s}_{\mathrm{c}}$, the individual slip events are comprised of a total of $N=$ 2 to 3 mobile dislocations, where $L\lesssim$ 0.5 μm according to Eq. (2). At the cut-off slip size ${s}_{\mathrm{c}}\approx$ 1$b$, we then find that ${N}_{\mathrm{c}}\approx$ 3 so that ${L}_{\mathrm{c}}\approx 0.7$ μm (Eq. (2)), which is in excellent accord with the deterministic value of the mean swept distance estimated in the above section. With increasing slip size ($s>{s}_{\mathrm{c}}$), single-ended source operation accounts for an increasing fraction of the total slip magnitude in the freestanding microcrystal. All of the observed slip events producing ${s>s}_{\mathrm{c}}$ then consist of $N\ge$ 3 mobile dislocations, reaching the maximum value of $N=$ 17 with $L$ $\approx 1.2$ μm for the largest measured avalanche event. The DDD simulations finally show that while dislocation storage and dislocation annihilation processes develop within the incipient slip regime, large avalanche emissions (${s>s}_{\mathrm{c}}$) are characterized by net dislocation storage (Fig. 4j).

The general slip distribution function in Eq. (3) is maintained for [100]-oriented Al microcrystals with $D=$ 8 µm, where ${s}_{\mathrm{c}}$ increases from $\approx$ 1$b$ to $\approx 3.5b$ indicating the onset of moderate dislocation interactions along with a further increase in ${L}_{\mathrm{c}}$ from $\approx 0.7$ μm to $\approx$ 2.3 μm (as described above). These microcrystals deform through bulk-like plasticity, which is evidenced by the occurrence of non-localized slip traces and sample barreling (Fig. 1a). Since the strain hardening response is further influenced by the dislocation stacking fault width, $d$, it is concluded that Al microcrystals with $d\approx$ 1 nm, thus exhibiting enhanced cross-slip and reduced interactions between mobile and forest dislocations, will tend to produce greater slip events than Cu microcrystals with $d\approx 3$ nm. According to the dislocation storage-recovery model, cross-slip would increase the estimated ${L}_{\mathrm{c}}$ by $\approx$ 15% in the Al crystals.

The following discussion concerns the scaling of the slip distributions as a function of the microsample size. In this sense, bulk-like microcrystal plasticity is known to fulfill a classic continuum mechanics description where the deformed state is prescribed by the shear strain $\gamma$ in the active slip systems. This implies that for a given slip event occurring in a hypothetical, representative material volume element, the number of mobile dislocations, $N$, increase in proportion with the element’s diameter, $D$, so that the same slip magnitude, $s$, is produced (Eq. (2)). Hence, the shear strain $\gamma =s/l$ is preserved irrespective of sample diameter $D$. Since a rather homogeneous slip distribution develops along the sample height $l$ (as evidenced from the diffuse slip traces in Fig. 1a), increasing microcrystal height gives rise to a proportional increase in the avalanche emission rate. In this sense, it is noted that in microcrystals deformed with the same $\dot{\varepsilon }$, a three-fold increase in $l$ raises $\nu (s)$ by the same three-fold factor (inset to Fig. 2). The shear strain $\gamma$ is consequently maintained irrespective of $l$. The aforementioned scaling between $N$ and $D$ obviously breaks down in sufficiently small samples where an incipient slip event (${s<s}_{\mathrm{c}}$) is already comprised of a single dislocation. This is inconsequential to our statistical analyses since the incipient slip domain adheres to a scale invariant power-law distribution.

Equations (2) and (3) prescribe that the avalanche distributions scale as

4 $$C\left(s\right)=A{s}^{-\kappa }{\mathrm{exp}}\left[-{\left(\frac{sD}{{bN}_{\mathrm{c}}{L}_{\mathrm{c}}}\right)}^{n}\right]$$

where the cut-off, mean dislocation glide distance ${L}_{\mathrm{c}}$ can be estimated through the measured stress–strain curves, as described above. As illustrated through the scaling of the slip distributions of Cu and Al microcrystals with different diameter $D$ via Eq. (4), see Fig. 2, it is found that the linear density, ${N}_{\mathrm{c}}/D$, of mobile dislocations carrying a slip magnitude ${s}_{\mathrm{c}}$ remains constant within the current range of $\rho \approx$10^14^ to 10^15^ m^-2^ where bulk-like microcrystal plasticity develops.

### Statistical transition towards confined plasticity in FCC microcrystals

Figure 2 contains the slip distribution from Cu microcrystals with the [100] orientation exhibiting a heterogeneous arrangement of localized slip traces along the sample height $l$. The stress–strain curves under such confined plasticity responses where dislocation network development is counterbalanced by surface annihilation are then characterized by the onset of large stress drops, $\Delta \sigma$, and $\theta =0$. Under these conditions, our results show that the cut-off slip increases from the value of ${s}_{\mathrm{c}}\approx$ 1 $b$, measured for the above bulk-like strain hardening microcrystals, to the current ${s}_{\mathrm{c}}\approx$ 12 $b$ in confined plasticity. As discussed next, this is consistent with the conception in that when strain hardening is hindered, plasticity is increasingly sustained by the activation of a limited number of single-ended sources in a steady, forest dislocation arrangement. The measured function $\nu (s)$ then shifts towards larger levels while dislocation glide becomes highly localized along the sample height (inset to Fig. 2).

The slip distributions from a second set of experiments in Cu microcrystals, tested as a function of the characteristic sample size $D$ and temperature (Fig. 3), are key to the statistical analysis of confined plasticity. These microcrystals also exhibit large $\Delta \sigma$ and the heterogeneous slip trace arrangement in Fig. 1b. The measured slip distributions indicate that when $D$ is reduced from 3.5 $\upmu$m to 1 $\upmu$m, a two-fold decrease in ${s}_{\mathrm{c}}$ is attained (see Fig. 3), which may only occur if the product ${\left(NL\right)}_{\mathrm{c}}$ exhibits a seven-fold decrease, as prescribed by Eq. (2). Even under the assumption that the revolution of single-ended sources about their pinning points in smaller microcrystals eventually leads to a smaller critical distance ${L}_{\mathrm{c}}$, the very significant seven-fold decrease in ${\left(NL\right)}_{\mathrm{c}}$ suggests that the number of mobile sources, ${N}_{\mathrm{c}}$, would also have to decrease when $D$ is reduced to $1\upmu$m. Along the lines of the dislocation mechanisms, the DDD simulations indicate that within the large avalanche domain, the total dislocation swept distance that is sustained through single-ended source operation becomes several times greater than the sample diameter (implying that $NL\gg D$). This is illustrated in Fig. 4g–i, where the pivoting of single-ended sources about multiple junctions with immobile dislocations results in the activation of dislocation segments at the intercepting cross-slip systems. Finally, following the thermally-activated nature of cross-slip, an increase in the total dislocation swept distance $(N{L)}_{\mathrm{c}}$ is anticipated to occur at elevated temperatures, as dislocation annihilation precludes stable junction formations and the mobile segments are disseminated across the active slip systems throughout the sample height. This is supported by the increase of the measured ${s}_{\mathrm{c}}$ and the attendant rise in $\nu (s)$ (see Fig. 3 for $D=$ 3.5 µm) along the onset of more noticeable localized slip (Fig. 1b). Increases in temperature thus facilitate the attainment of confined plasticity.

As the sample diameter decreases further towards submicrometer sizes (i.e. $D\approx 0.6$ µm in Fig. 3), the slip distributions exhibit drastic reductions in ${s}_{\mathrm{c}}$ and $\nu (s)$ which are attributed to severe dislocation source starvation. Therefore, the marked decrease in the number of mobile dislocations, ${N}_{\mathrm{c}}$, in the transition from micrometer to submicrometer sizes significantly reduces the product ${\left(NL\right)}_{\mathrm{c}}$, which results in the currently measured smaller ${s}_{\mathrm{c}}$ values. This is supported by the DDD simulations performed with $D=1$ µm, where only one single-ended source recursively operates ($\rho =$ ${10}^{12}$ m^-2^; $N=1$) under a fixed value of the shear stress, resulting in markedly serrated stress–strain curves and a similar ${s}_{\mathrm{c}}\approx 4b$ as in the above experiments with $D\approx 0.6$ µm (see Fig. 4e,f). These simulations eventually show the onset of cross-slip events of the active source and the occurrence of collinear interactions due to the presence of a few secondary dislocation segments. The latter leads to the segmentation of the slip events, which reduces ${s}_{\mathrm{c}}$.

Assessment of the correlation between the magnitude of the slip events and the applied shear stresses in the active slip systems is finally illustrated in Fig. 2b,c. We choose to evaluate this feature through Pearson correlation coefficient, $r$, rising towards unity when an increasingly linear relationship is reached between the scattered slip population and the stress, and the $p$-value prescribing the likelihood in that this relationship is merely coincidental. In the FCC microcrystals in Fig. 2c, it is found that the same scattered slip distribution develops irrespective of the applied stress level, a feature prescribed by small $r<$ 0.2 or by large $p>$ 20% values. This characterizes the development of bulk-like plasticity both under intense and moderate dislocation interactions, where the size of the individual avalanche events is statistically unaffected by the applied stress, along the lines of SOC. It is then noted that the greatest $r$ values from our analysis pertain to the smaller microcrystals deforming well within the confined plasticity regime, where $r$ lies in the 0.3 to 0.4 range while $p<$ 5%. These values suggest the onset of stress dependent slip distributions, where the likelihood for large avalanche emissions ($s>{s}_{\mathrm{c}}$) correlates with the application of large shear stresses that are greater than a critical ${\tau }_{\mathrm{c}}$ value (see Fig. 3b). Therefore, the $C(s)$ distributions measured at larger stress levels would tentatively exhibit a higher ${s}_{\mathrm{c}}$, in support of STC.

Along the above analyses, it is noted that a single displacement-controlled *experiment* does not provide a significant number of slip events within a window of stress so as to enable assertion of the possible stress-binning of function $C(s)$. Therefore, it is not statistically sound to evaluate whether the avalanches adhere to STC or SOC by comparing between the averaged slip magnitudes attained within different windows of stress. The variability in the stress level leading to sustained plastic flow in different (nominally identical) microsamples further prevents such assertions from a single dataset containing all slip events from these experiments.

### Mechanisms and statistics of intermittent plasticity in BCC microcrystals

**Figure 5:**
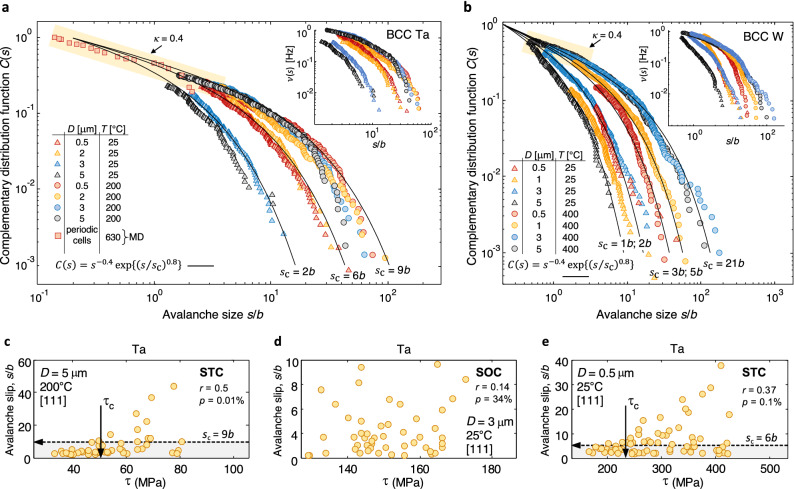
Master distributions of the individual slip events from the experiments and MD simulations in BCC Ta (**a**) and BCC W (**b**), where the cut-off slip size *s*_c_ marks the transition from incipient slip to the large avalanche domains prescribed through the *C*(*s*) function in Eq. (3). At RT, reductions in micropillar diameter *D* lead to greater *s*_c_. The reversed trend is found in (**b**) at increasing temperatures. This is associated with the onset of dislocation cross-kinking along with a transition from planar to wavy glide. The insets provide emission frequency function *ν*(*s*) from the experiments in the main figures. Evolution from STC to SOC is illustrated in (**c**,**d,e**). See text for detailed discussions.

**Figure 6:**
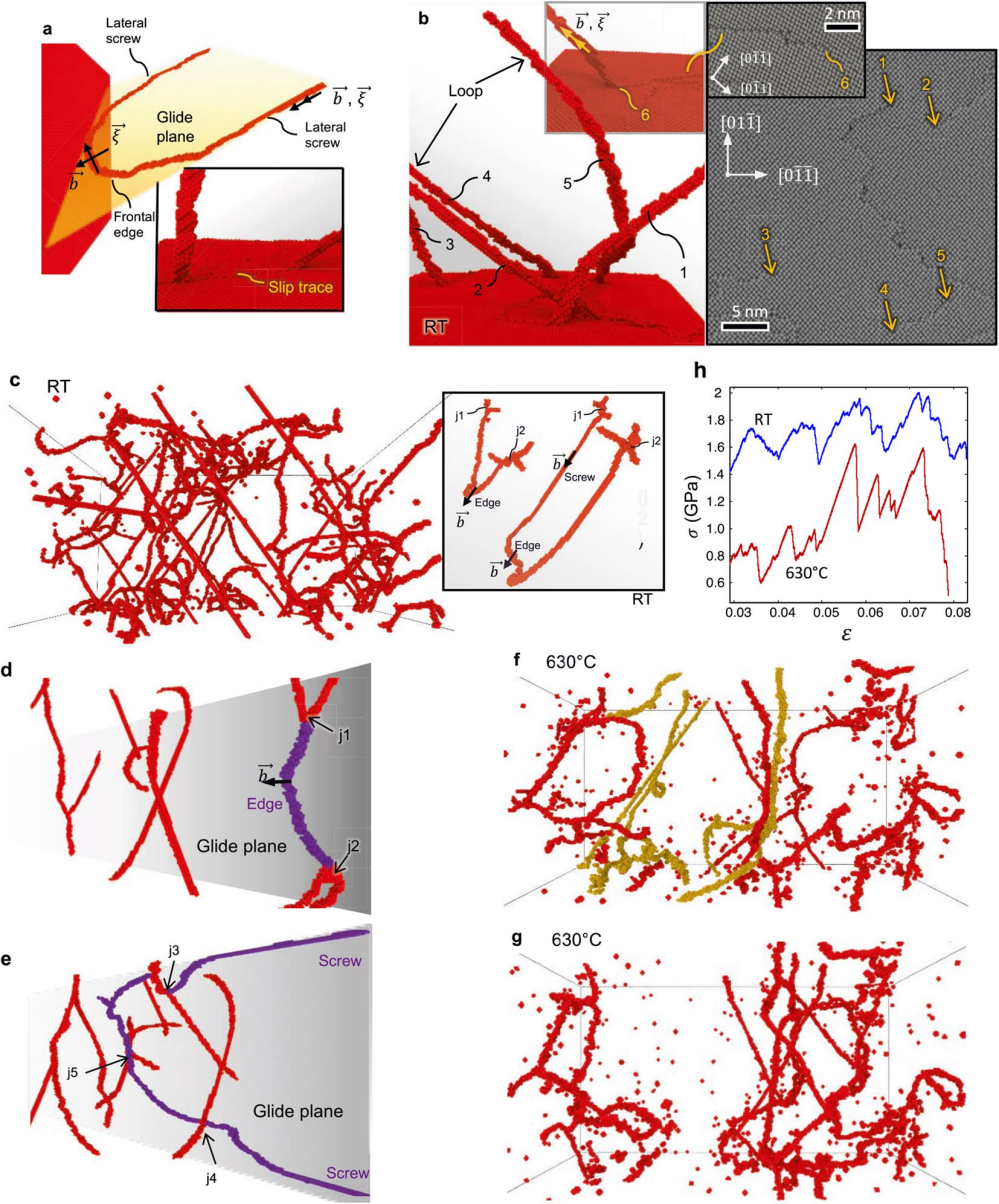
Avalanche propagation and slip trace emissions from the MD simulations in BCC Ta. (**a**) Anisotropic loop expansion due to the preferential glide of frontal edge segments while lateral partnering screws remain rather immobile. The inset shows associated (straight) slip trace formation. (**b**) Development of wavy slip traces as meandering dislocation segments (1, 2, 3) and dislocation loops (4, 5), detached from the entangled dislocation network, annihilate at the surface. The inset shows the locally wavy traces produced by sweeping of a surface intercepted screw segment (6). (**c**) Characteristic RT dislocation network comprised by long screw segments. The inset illustrates the anisotropic loop expansions from which the entangled network emerges. (**d**) Avalanche produced by the expansion of a dislocation loop (violet) pinned at j1 and j2. The loop is arrested in (**e**) due to the interaction with the immobile network (red) at points j3, j4 and j5, see online Supporting Movie S2. (**f**,**g**) Avalanche emitted at 630 °C where significant cross-kinking occurred. The figures illustrate the activation of the dislocation segments in green (*ρ* ≈ 5 × 10^15^ m^−2^) which vanish in (**g**) as they are mutually annihilated, see online Supporting Movie S3. (**h**) Stress–strain curves at RT and 630 °C, where statistically greater stress drops occur at elevated temperatures.

The slip distributions in BCC microcrystals, shown in Fig. 5, adhere to the general function in Eq. (3), where the Kolmogorov–Smirnov test confirms the power-law hypothesis with unique $\kappa$ exponent $\approx$ 0.4 $\pm 0.06$ $({\lambda }_{1}\approx$ 1.4) for ${s<s}_{\mathrm{c}}$. In the light of Fig. 1c and the dislocation interaction and slip trace formation mechanisms revealed in Fig. 6, it is argued in the following discussion that the measurement of different ${s}_{\mathrm{c}}$ values in BCC microcrystals is the signature of the distinct mobility of the screw dislocations as a function of temperature, sample size and the applied stress level.

In submicrometer-sized pillars deformed at RT, plastic deformation proceeds through single-ended source operation^26,27^ at applied stresses reaching the distinctive large Peierls barrier^48–50^. Plasticity thus sets-in at $\sigma \gtrsim$ 2000 MPa, as shown in Fig. 1c for *D *$\le 0.5$ µm, where the character of the mobilized single ended-sources alternates from pure edge to pure screw (inset to Fig. 1c). It is then argued that the recurrent cross-kinking of screw segments triggered under such large applied stresses^51^ leads to the wavy slip trace emissions observed in the W microcrystals (see Fig. 1c where similar slip patterns arise in the RT tests).

When $D\gtrsim$ 3 μm, full dislocation loops can be effectively accommodated within the microsample. At RT, the MD simulations suggest that the plastic intermittencies are produced under the smaller applied stresses yielding anisotropic loop expansions along the highly mobile edge segments of the loops (Fig. 6d,e). Although the slip traces remain globally straight (Fig. 1c), atomistically wavy patterns arise through cross-kinking mechanisms of the surface-intercepted screw segments (Fig. 6a). It is noted that while the enhancement of cross-kinking at large applied stresses^51^ is consistent with the observed wavy glide in the W microcrystals (3 μm $<D<$ 5 μm) deformed at RT, where $\sigma \approx$ 1500 MPa, planar glide was found to prevail in the Ta microcrystals of similar size that exhibited plastic deformation at the smaller stresses, $\sigma \approx$ 300 MPa, where cross-kinking is hindered.

A highly dense dislocation network may also develop in the larger microcrystals ($D\approx$ 5 μm), as modelled through the MD simulations with periodic boundaries that mimic bulk-like responses. This RT dislocation network is characterized by long screw segments (Fig. 6c) in accord with experimental findings in macroscale samples^48,52^. The MD simulations with free-standing cells that contain similar entangled dislocation networks then predict the predominance of nanoscale, wavy slip features as the meandering mobile dislocation segments released from the dislocation structure intercept the surface (Fig. 6b). Although these wavy glide features facilitate slip trace dissemination throughout the sample length along with the activation of cross-kinking in the W microcrystals deforming at large stresses ($\sigma \approx$ 1500 MPa and $D\approx$ 5 μm), the localization of these wavy deformation features within individual slip traces results in the planar deformation mode that was experimentally observed in the Ta microcrystals of similar size, where plasticity was triggered at the smaller $\sigma \approx$ 300 MPa *in the absence of cross-kinking*. The development of planar, localized glide has been observed in *bulk* BCC crystals^53^ which also exhibit the smooth stress–strain curves in Fig. 1c for $D\approx$ 5 μm. Therefore, slip localization is not necessarily associated with confined plasticity.

Concerning the statistical analysis of the slip distributions, Fig. 5 shows the onset of higher ${s}_{\mathrm{c}}$ and $\nu (s)$ in small BCC microcrystals (0.5 μm $<D<$ 2 μm) where plasticity is sustained at the *large* applied stresses in Fig. 1c. This implies the attainment of a larger, characteristic total dislocation swept distance (which is statistically measured through the product ${(NL)}_{\mathrm{c}}$) as compared to in microcrystals with $D\approx$ 5 μm, deforming under *smaller* stresses. It is noted that the larger ${s}_{\mathrm{c}}$ in the smaller microcrystals is not a direct outcome of the reduction in the diameter $D$ (prescribed by the relation ${s}_{\mathrm{c}}=[b{\left(NL\right)}_{\mathrm{c}}/D]\propto 1/D$), as the measured three-fold increase in ${s}_{\mathrm{c}}$ resulting under the maximum ten-fold decrease in $D$ can only occur under a significant decrease of the product ${(NL)}_{\mathrm{c}}$). Finally, *smaller* ${s}_{\mathrm{c}}$ values are measured within the range of $D<$ 3 μm when the activation of cross-kinking and the onset of wavy glide become more evident (cf. RT experiments where $D$ ranges from 2 to 3 μm in Fig. 5a for Ta—planar glide at larger ${s}_{\mathrm{c}}$—with Fig. 5b for W—wavy glide at smaller ${s}_{\mathrm{c}}$). At fixed microcrystal size, cross kinking is thus associated with a reduction in ${(NL)}_{\mathrm{c}}.$

The MD simulations reveal that irrespective of the testing temperature, the incipient slip domain is governed by the gliding of individual screw and edge dislocation segments over a mean free path length $L$ of more than 5 atomic lattices, yielding $s\gtrsim 0.2b$. The distinctive features of the high temperature MD simulations are the increased waviness of the screw segments (cf. Fig. 6c,f) owing to the thermally and stress-activated nature of kink-formation and cross-kinking phenomena^48,51^, as well as the marked rise in the velocity of the mobile dislocations. The large avalanche domain is then associated with the gliding of several dislocation segments along predominantly edge sides, a feature that is facilitated by the enhanced mobility of the lateral, partnering screws. The edge segments thus sweep across $L$ values approaching a hundred of atomic lattices, where the activation of the first dislocation segment triggers gliding of secondary segments. Moreover, the dislocation entanglement is reduced during high temperature avalanche emissions as the mobile segments become mutually annihilated (cf. Fig. 6f,g along with the Supporting Movies S2 and S3). Large stress drops are consequently produced (Fig. 6h) where avalanche arrest occurs in less entangled arrangements that withstand smaller applied stresses. The above results are consistent with the experimentally measured increase in the cut-off slip magnitude ${s}_{\mathrm{c}}$ in the high temperature avalanche distributions, along with a surge of large avalanche emissions as evidenced through the measured larger $\nu (s)$ (see the Fig. 5a,b). It is thus argued that the high temperature avalanche distribution is characterized by greater ${N}_{\mathrm{c}}$ and ${L}_{\mathrm{c}}$ values which markedly increase parameter ${(NL)}_{\mathrm{c}}$.

The influence of sample size on the slip distribution is less clear at elevated temperatures (Fig. 5a), where even a reversed trend is found indicating the occurrence of larger slip events (characterized by higher ${s}_{\mathrm{c}}$ values) as the microcrystal size is increased (Fig. 5b). To rationalize these results, it is important to consider that a thermally-assisted transition from homogeneous (wavy glide) to heterogeneous (planar glide) slip trace distributions is found in BCC W with increasing microcrystal size (Fig. 1c). The wavy glide features observed in the *smaller* W microcrystals ($D<$ 1 μm) are then consistent with the onset of cross-kinking at the *large* applied stresses ($\sigma >$ 2000 MPa) needed to sustain plastic deformation for $T=400$ ºC ($T/{T}_{\mathrm{m}}$ $\approx 0.11$). On the other hand, planar glide prevails in the *larger* W microcrystals ($D\approx$ 5 μm) where cross-kinking is hindered under *smaller* applied stresses ($\sigma <$ 1000 MPa). In accord with the above discussion for the RT experiments where planar glide is associated with a higher ${s}_{\mathrm{c}}$, the larger W microcrystals tested at elevated temperatures then exhibit higher ${s}_{\mathrm{c}}$ and ${(NL)}_{\mathrm{c}}$ values than the smaller microcrystals (Fig. 5b). Finally, the role of temperature in facilitating large avalanche emissions also becomes evident when comparing the slip distributions from Ta and W microcrystals that exhibited similar planar glide responses (cf. Fig. 5a,b for $D>$ 3 μm). Higher ${s}_{\mathrm{c}}$ and ${(NL)}_{\mathrm{c}}$ are thus measured in the W microsamples tested at a greater homologous temperature ($T/{T}_{\mathrm{m}}$ $\approx$ 0.11) than the Ta counterparts ($T/{T}_{\mathrm{m}}$ $\approx 0.07$).

Our RT experiments finally show that function $C(s)$ is increasingly binned by stress with decreasing sample size (cf. Fig. 5d,e), so that larger avalanches are emitted when the applied stress is greater than some critical level ($\tau >{\tau }_{\mathrm{c}}$). Since this feature gradually vanishes in the larger BCC microcrystals with small ${s}_{\mathrm{c}}$ values, the slip distributions tentatively exhibit a transition from STC to SOC. Increasing temperature then results in statistically larger avalanches for $\tau >{\tau }_{\mathrm{c}}$, supporting the prevalence of STC (Fig. 5c).

## Concluding remarks

Prior investigations of plastic intermittencies involved the large strain bursts triggered with dynamically-reactive testing systems. Under these driving conditions, the microcrystal is rapidly compressed as a function of the system’s dynamic response, which ultimately governs the termination of the instability and its overall size. We argue that a dynamically-induced plastic instability or strain burst is comprised of many of the currently measured individual dislocation avalanche events, propagating over minuscule time scales, which are ubiquitous to crystal plasticity. Our investigation provides in-depth statistical and mechanistic understandings to these individual ultrafast dislocation avalanche events, extending our knowledge of intermittent plasticity.

The following are the salient conclusions from our work:

1. Our experiments and simulations consistently show that the size distribution of the individual dislocation avalanche events is characterized by a power-law, incipient slip regime which spans over up to 2 decades of plastic slip sizes ($s$ $\approx$ 0.1$b$–20$b$). The regime of incipient slip adheres to the same universal power-law exponent $\lambda \approx$ 1.4–1.5 that was reported in the previous investigations of strain bursts^3,8^. It is comprised of multiple dislocation segments that simultaneously glide at numerous locations in the microcrystal without significant dislocation density build-up.
2. A large avalanche domain with reduced emission probability sets-in upon the truncation of the incipient slip regime at the cut-off slip size ${s}_{\mathrm{c}}$, as prescribed though the universal avalanche distribution function in Eq. (3). It is shown that when the individual dislocation avalanche events are recorded, the cut-off slip ${s}_{\mathrm{c}}$ becomes the essential parameter prescribing the influence of crystalline structure, testing temperature and microsample size—*vis-à-vis* the onset of collective dislocation glide, dislocation interaction and surface dislocation annihilation mechanisms—in intermittent crystal plasticity. This provides insight into the role of the underlying slip phenomenology upon the dislocation avalanche distribution^14,54–59^.
3. Bulk-like plasticity in FCC Al and Cu microcrystals is characterized by a homogeneous slip trace distribution along with sample barreling. Large avalanche propagation then results in the storage of dislocation segments through junction forming processes. In continuum microcrystal plasticity analyses, the cut-off slip ${s}_{\mathrm{c}}$ attains through the emission of a slip event comprised of ${N}_{\mathrm{c}}$ mobile dislocations which sweep across a mean distance ${L}_{\mathrm{c}}$ in a sample of diameter $D$. Since ${s}_{\mathrm{c}}=b{(NL)}_{\mathrm{c}}/D$, a statistically sound measure to the mean dislocation swept distance is obtained through the characteristic slip size ${s}_{\mathrm{c}}$ of the distribution. This distance is the key continuum crystal plasticity parameter to the assessment of dislocation junction formation and strain hardening within the seminal dislocation storage-recovery model^45^. In the absence of a marked increase of the dislocation density $\rho$ with plastic straining, as in the case of the current microcrystals with moderate $\theta$, the avalanche distributions exhibit fixed ${s}_{\mathrm{c}}$ and ${L}_{\mathrm{c}}$. Since the cut-off slip ${s}_{\mathrm{c}}$ is then independent of the effective shear stress $\tau$ in the active slip systems, intermittent plasticity becomes a spontaneous self-organized process, in support of SOC. It is emphasized that while ${L}_{\mathrm{c}}$ and ${s}_{\mathrm{c}}$ would gradually *decrease* if plastic straining involved pronounced dislocation storage at increasing applied stresses ($\theta \gg$ 0), this behavior clearly challenges the distinctive *increase* in ${s}_{\mathrm{c}}$ triggered at high stresses under STC and confined plasticity, as summarized next.
4. Pure FCC microcrystals that deform under confined plasticity exhibit greater values of the cut-off slip ${s}_{\mathrm{c}}$ along with a transition from uniformly distributed to highly localized slip trace patterns. Confined plasticity attains under insufficient dislocation interactions, so that network development is hindered by surface dislocation annihilation when ${L}_{\mathrm{c}}>D/2.$ A marked increase in the total distance travelled by the mobile dislocations is observed due to recurrent single-ended source operation, resulting in higher values of the product ${(NL)}_{\mathrm{c}}$. Our experiments and DDD simulations further show that when the sample size is decreased towards the submicrometer scale, severe dislocation source starvation leads to a drastic reduction in ${s}_{\mathrm{c}}$. The natural scaling of the number of mobile dislocations with microcrystal diameter, characterizing continuum bulk-like crystal plasticity thus breaks down. Our experimental results suggest that the onset of the large avalanche domain under confined microcrystal plasticity is governed by the shear stress in the active slip systems. Since greater slip events result from single-ended source operation in conjunction with dislocation network destabilization processes at increasing applied stresses, a *higher* ${s}_{\mathrm{c}}$ is measured when $\tau$ surpasses a critical ${\tau }_{\mathrm{c}}$ level, in support of STC. The parameter ${L}_{\mathrm{c}}$ then becomes a function of the microsample size. It is finally noted that while the homogenization of the slip trace distribution is favored under wavy glide, the suppression of cross-slip in alloyed FCC metals that exhibit planar glide^60^ and moderate $\theta$ facilitates slip localization, arguably triggering the onset of confined microcrystal plasticity.
5. The slip distributions in BCC microcrystals are governed by the sluggish glide of the screw dislocation segments. In spite of the markedly different BCC dislocation glide phenomenology, it is found that the range of ${s}_{\mathrm{c}}$ values measured under bulk-like BCC plasticity ($D\gtrsim$ 5 μm) are similar to those from FCC counterparts exhibiting marked dislocation interactions. *Larger* values of the cut-off slip ${s}_{\mathrm{c}}$ are then observed in the *smaller* BCC microcrystals, where plasticity is triggered through single-ended source operation under the large shear stress level surpassing the Peierls barrier of the screw segments. This indicates an increase of the product ${(NL)}_{\mathrm{c}}$. Moreover, a transition from planar to wavy slip is eventually observed in the smaller microsamples, denoting the onset of cross-kinking mechanisms. This is characterized by the decrease of the cut-off slip ${s}_{\mathrm{c}}$ and the product ${(NL)}_{\mathrm{c}}$. Finally, a pronounced raise of ${s}_{\mathrm{c}}$ is observed at elevated temperatures, evidencing an increase in the number of mobile dislocation segments, ${N}_{\mathrm{c}}$, in conjunction with a higher ${L}_{\mathrm{c}}$. This is in accord with the enhanced mobility of the screw segments observed in the high temperature MD simulations. In the line of the above slip phenomenology, the occurrence of planar glide at high temperatures facilitates the onset of stress-dependent avalanche distributions, where higher ${s}_{\mathrm{c}}$ and ${L}_{\mathrm{c}}$ are promoted for $\tau >{\tau }_{\mathrm{c}}$, in support of STC.

## Supplementary information

Supplementary Information.

Supplementary Video 1.

Supplementary Video 2.

Supplementary Video 3.
