# Supplementary material for: Statistics of dislocation avalanches in FCC and BCC metals: dislocation mechanisms and mean swept distances across microsample sizes and temperatures
Source: Sci Rep. 2020 Nov 4;10:19024. doi: 10.1038/s41598-020-75934-5 (PMC7642400; doi:10.1038/s41598-020-75934-5)
Supplement: Supplementary file 1 — Supplementary Information. [file 41598_2020_75934_MOESM1_ESM.docx]

**Appendix to the manuscript “Statistics of dislocation avalanches in FCC and BCC metals: dislocation mechanisms and mean swept distances across microsample sizes and temperatures”**

Jorge Alcalá^1^, Jan Očenášek^2^, Javier Varillas^1,2^, Jaafar El-Awady^3^,

Jeffrey M. Wheeler^4,5^, Johann Michler^5^

^1^ Department of Materials Science and Metallurgical Engineering, InSup, ETSEIB. Universitat Politècnica de Catalunya, 08028 Barcelona, Spain

^2^ New Technologies Research Centre, University of West Bohemia in Pilsen, 30614 Plzeň, Czech Republic

^3^ Department of Mechanical Engineering, Whiting School of Engineering, The Johns Hopkins University, Baltimore, MD 21218, USA

^4^ Laboratory for Nanometallurgy, Department of Materials, Swiss Federal Institute of Technology in Zürich, Vladimir-Prelog-Weg 5, CH-8093 Zürich, Switzerland

^5^ Laboratory for Mechanics of Materials and Nanostructures, Empa, Swiss Federal Laboratories for Materials Science and Technology, 3602 Thun, Switzerland

***1. Testing systems and the measurement of stress and displacement-controlled driven avalanches***

The boundary conditions of the testing system impose large constraints on the mechanical behavior of micro-scale samples. In a dynamically-reacting system, the cross-head/flat punch indenter tip is driven by an electromagnetic or electrostatic actuator. Along the load train, this actuator’s solenoid acts as the dynamic mechanical analogue of a spring. When a plastic intermittency occurs under *load control*, the flat punch indenter is rapidly displaced forward until sufficient resistance is encountered at the specified load level through the advent of strain hardening or dislocation source exhaustion in the micro-sample. As shown in Figure A1(a) for microcrystals with diameters in the size range of several microns, this can be equivalent to strains of 2-5% for a single burst spanning over a time frame of $\sim$ 50 ms. More advanced dynamically reactive systems with effective time constants in the range of a hundred of microseconds may reduce the size of the strain bursts with faster system dynamics [1]. When these systems are operated under *extrinsic displacement control*, the rapid forward motion of the tip triggered by the plastic intermittency is eventually restrained after a burst by a feedback loop, which actuates upon the relaxation of the system dynamics. The plastic strain accumulating in the largest bursts then becomes in the 1-2% range. The displacement overshoot of the tip position during the strain burst is then subtracted by the electronic control of the testing system, which reduces the force applied to the solenoid and unloads the micro-sample until the displacement rate returns back to the feedback loop’s set displacement rate.

In the currently employed system operating under *intrinsic* displacement control, a stiff piezoelectric-actuated cross-head drives tip penetration. The load cell element acting as a spring is then located at the opposite side of the load train (i.e., next to the micro-sample holder). This setup allows the cross-head to maintain its current position, regardless of any plastic intermittency occurring in the micro-sample. All intermittencies, which result in stress drops, develop at a fixed indenter tip displacement, while the sample releases elastic stored energy into the load cell spring without the possibility of causing additional plastic deformation. It is currently found that the size distribution of the stress drops becomes a signature of intermittent plasticity irrespective of the system dynamics or feedback loop. In an experiment operated under *intrinsic* (strict) displacement control, hundreds of stress-drops can then be counted within the 1% strain traversed during a single *extrinsically-controlled* strain burst. The characteristic stress drops and the currently implemented automatic counting scheme are illustrated in Figs. A1 (b) and (c).

According to our MD simulations, a medium sized avalanche with $s=$ $2b$ driven under strict displacement control propagates at $\sim$0.1 km/s. To put this result into perspective, it is noted that while the free-flight of a dislocation may become supersonic ($>$1 km/s) when subjected to large applied stresses [3], the velocity of a dislocation avalanche is necessarily reduced as the mobile dislocations (i) revolve around their pinning points, (ii) become stored within the forest (immobile) dislocation arrangement, and (iii) dislocation activity is triggered in the forest segments so as to produce further avalanche propagation. Since the sweeping of the above avalanche across a microcrystal with $D=2$ μm requires $\sim$ 20 ns, the total time spent in the propagation of the $\sim$ 100 medium-sized avalanches encompassed within a 1% plastic strain would be $\sim$ 2 μs. This is $\sim$ 4 decades smaller than the total time interval over which an *extrinsically-driven* strain burst traversing the same 1% strain is bound to occur. A key aspect from the MD results is that essentially strain-rate independent stress drops are attained even when the sample is deformed at the extreme, externally applied strain rates of 2$\times{10}^{6} s^{-1}$. Notice that this is many decades greater than the effective strain rate imposed during the onset of an extrinsically-controlled strain burst.


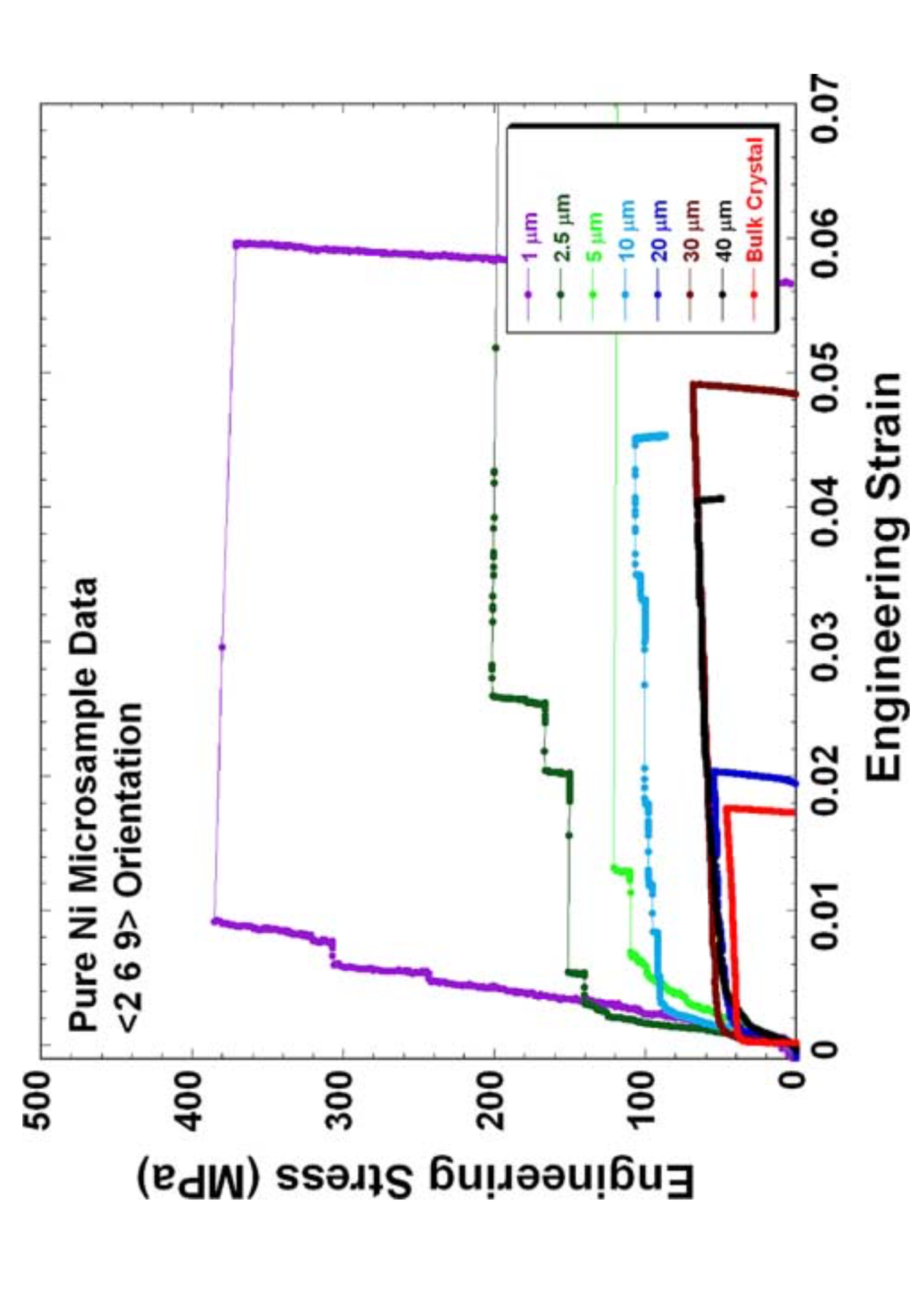
*
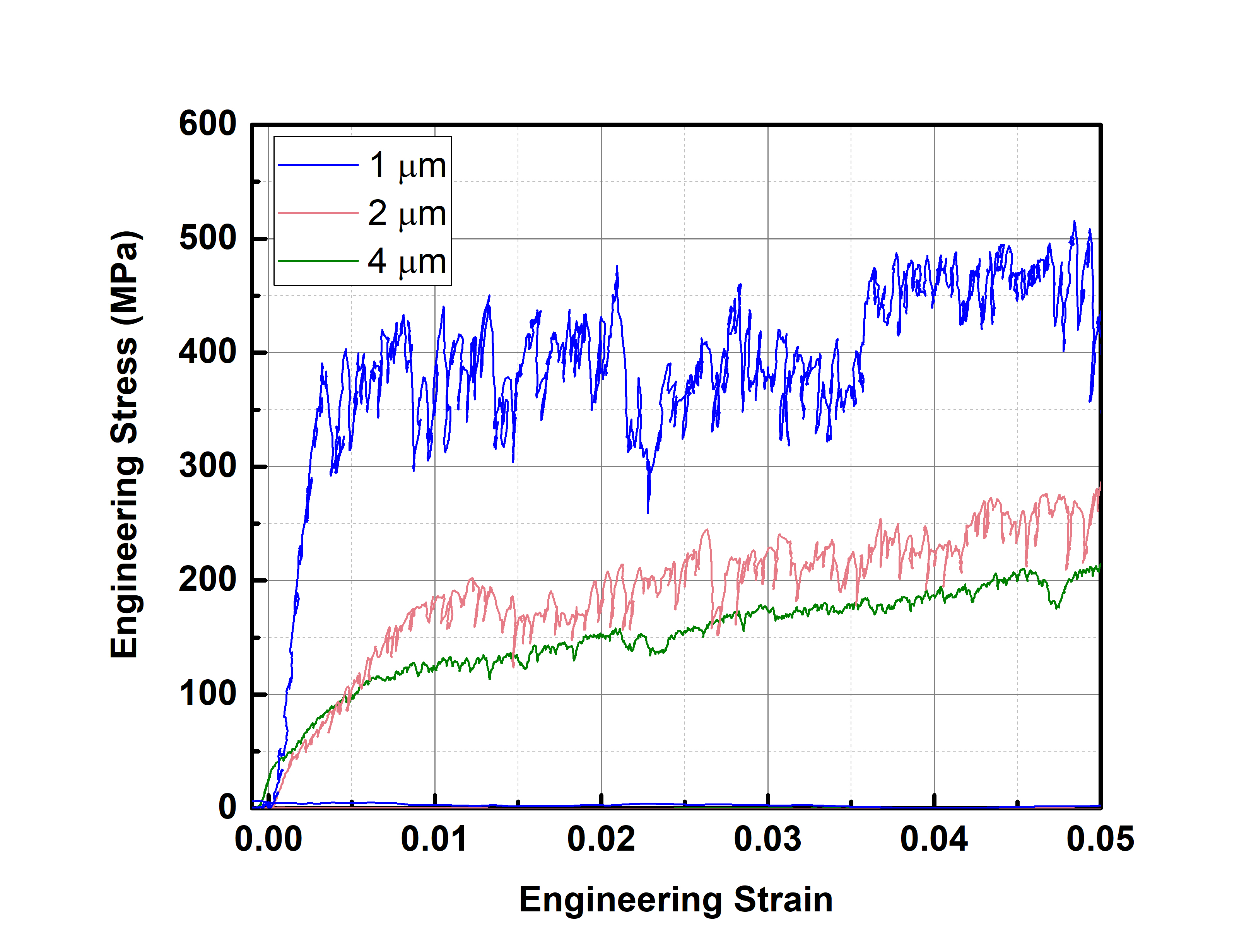
*

(a)

(b)

**Pure Ni
<627> Orientation**

*
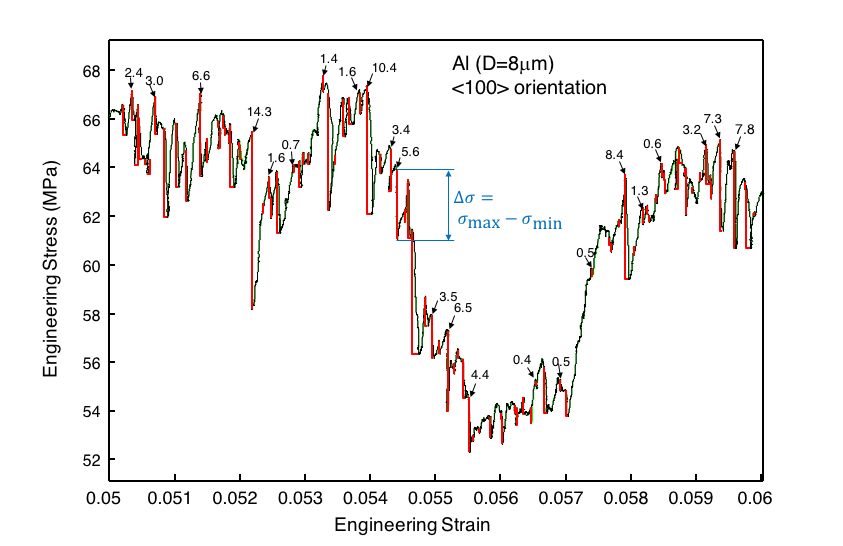
Fig. A1: Comparison between strain bursts observed in (a) with a dynamically-reactive system under load-control [2] and (b) current intrinsic displacement-controlled compression of single crystalline pure nickel micropillars oriented for single-slip. A similar plot as in (a) is observed for experiments conducted under extrinsic displacement control with dynamically-reactive systems, even though the size of the strain bursts is reduced. Details of the stress serrations from a micropillar compression experiment in Al, performed under intrinsic displacement control, is shown in (c) within a plastic strain range of 1%. The individual dislocation glide events associated with each stress drop* $\Delta\sigma$ *are marked in red in (c). [These stress drops are currently counted when their magnitude exceeds the load cell resolution by 3 times.] A number of the underlying plastic slip magnitudes are also given in (c) in multiples of the Burgers vector. It is emphasized that all individual intermittent glide events in (b) and (c) are lost when the micro-samples are strained with dynamically-reactive systems (see text for details).*

(c)

The above analysis suggests that the strain bursts produced with dynamically reactive systems are indeed constituted of many stress drops, essentially occurring at fixed displacement over minuscule time scales. The following scenario can be therefore envisaged during the onset of a dynamically-driven burst. As the first almost instantaneous dislocation avalanche event occurs, the micro-sample is effectively subjected to the stress drop $\Delta\sigma=\sigma_{\max}-\sigma_{\min}$ (see Fig. A1(c)). With this loss of restraining stress, the testing system reacts by accelerating the mass of the solenoid/indenter tip at the applied force to further deform the micro-sample. A large number of *individual* dislocation avalanche events, each characterized by their own $\Delta\sigma$, then occur unnoticed during the forward motion of the tip. It is argued that the propagation of all such individual avalanches is effectively quasi-static as the indenter tip can be regarded to maintain a fixed position during the aforementioned minuscule avalanche propagation time interval of $\sim$ 20 ns. The strain burst is finally terminated when the micro-sample sustains an applied stress greater than $\sigma_{\max}$ (for a dynamic system operated under load control) or when the total strain burst becomes sufficiently large (for a dynamic system operated under *extrinsic* displacement control). It is noted that in both events, sufficient time needs to accumulate so that the forward motion of the indenter tip can be effectively stopped by the feedback loop.

The above straining conditions contrast with those occurring in tests driven under *intrinsic* (strict) displacement control, where the recording of each individual stress drop, $\Delta\sigma$, is enabled irrespective of system dynamics. In this sense, it suffices to note that the indenter tip motion is independent of any avalanche events. Beyond some dynamic relaxation of the load train, the recorded stress is then bounded to reach the effective $\sigma_{\min}$ level. The smooth forward (quasi-static) motion of the indenter tip is maintained throughout the entire microcrystal compression experiment, where the externally-applied strain rates lie in the range of $\dot{\varepsilon}\sim{10}^{-3}-{10}^{-4}$ s^-1^.

***2. Assessment of the avalanche slip magnitudes***

As illustrated in Fig. A1(c) and in the inset to Fig. 1(c) from the main text, an avalanching event is characterized by the stress drop $\Delta\sigma=\sigma_{\max}-\sigma_{\min}$ and by the associated plastic slip magnitude $s$. When a stress drop strictly develops at fixed displacement, the accumulated uniaxial *plastic* strain becomes equal to the relaxed elastic strain induced during sample unloading from $\sigma_{\max}$ to $\sigma_{\min}$. Since the axial displacement $\Delta u=s \times SF$ produced by the stress drop $\Delta\sigma$ is then equal to the elastic relaxation, it follows that

$$\frac{\Delta\sigma}{E}=\frac{\Delta u}{l}= \frac{s \times SF}{l} (A1)$$

where $SF$ is the Schmid factor and $s$ is the plastic slip magnitude. Hence,

$$s=\left[ \Delta\sigma l/(E \times SF) \right] . (A2)$$

Fig. A1(c) illustrates the avalanche counting and the associated slip levels obtained from the stress drops in *intrinsic* (strict) displacement-controlled experiments (Eq. (A2)). The minimum discernable slip magnitude, $s_{\min}$, therefore depends on the noise level of the load cell employed in the microcompression system, which in our case is 3 $\mu$N RMS. The minimum measurable slip event is thus set at three times of the load cell resolution.

When an individual slip event propagates through a certain time interval, the externally applied strain rate has to be sufficiently small as to neglect the onset of any additional uniaxial strain $\Delta\varepsilon$ (see Fig. A2). In this sense, it is emphasized that for the strain rates applied in the MD and DDD simulations, the slip magnitudes $\Delta s$ accounting for strains $\Delta\varepsilon$ in Fig. A2 become smaller than 20% of the associated slip and strain magnitudes, $s$ and $\varepsilon$. Under these conditions, the inferred values of $\kappa_{1}$, $\kappa_{2}$ and $s_{c}$ are essentially unaffected by such slip and strain increments, as illustrated in Fig. A2(a).

Assessment of the shear stress $\tau^{(\alpha)}$ is then performed through $\tau^{(\alpha)}=\sigma\mathrm{Cos}\theta\mathrm{Cos}\lambda$, where the angles $\theta$ and $\lambda$ define the orientation of the slip system $\alpha$ with respect to the externally applied, normal stress $\sigma$. Experimental support for the activation of the slip system with the highest shear stress is then obtained from the localized slip traces characterizing confined microcrystal plasticity, from which the active slip plane can inferred through detailed Scanning Electron Microscopy (SEM) observations.

Since our simulations show that the characteristic time frame for avalanche propagation is extremely small, the slip and the strain increments, $\Delta s$ and $\Delta\varepsilon$, developing in our *experiments* cannot truly occur *during* the propagation of the avalanches themselves. It then follows that these slip and strain increments become the unique signature of the dynamic relaxation occurring in the testing system during each stress drop, $\Delta\sigma$. This is consistent with the clear reduction in the measured magnitudes of $\Delta s$ and $\Delta\varepsilon$ which results as the currently experimentally applied small strain rates are reduced from $\dot{\varepsilon}= {10}^{-3}$ s^-1^ to ${10}^{-4}$ s^-1^ (where all $\Delta s$ values again become clearly smaller than 20% of the slip magnitudes $s$ accumulating during each stress drop $\Delta\sigma$).

***3. Computation of the slip distributions, power-law exponents and definition of the critical slip event size*** $\boldsymbol{s}_{\boldsymbol{c}}$

The cumulative distribution, $D\left( s \right)$, of a continuous probability density function, $P(s)$, is defined as

$$D\left( s \right)=\int_{s_{\min}}^{s} P\left( s \right)ds , (A3)$$

where $P\left( s \right)=0$ for $s<s_{\min}$ and $\int_{0}^{\infty} P\left( s \right)ds\equiv1$ . Since $P\left( s \right)=As^{-\lambda}$, $A$ becomes the normalization constant fulfilling the above integral.

(a)

(b)

*Fig. A2: Stress drops marking avalanche emission and propagation. The results are from the MD simulations in BCC Ta at RT. Part (a) shows the* $C(s)$ *distribution calculated through Eq. (A1) exclusively accounting for the strain,* $\varepsilon$*, carried by an individual slip event, as well as by adding the additional strain,* $\Delta\varepsilon$*, produced by the displacement,* $\Delta s$*, produced during the stress drop. Part (b) shows strains* $\varepsilon$ *and* $\Delta\varepsilon$ *occurring at different displacement rates, where the latter is smaller than 20% of the former when the computational domain is deformed at 0.1 m/s.*

The complementary cumulative distribution function, $C(s)$, prescribes the emission probability of all slips greater than a certain $s$ value ($s>s_{\min}$), and is therefore given by

$$C\left( s \right)=\int_{s_{\min}}^{\infty} P\left( s \right)ds . (A4)$$

Since $\lambda>1$, it follows that $C\left( s \right)=\frac{A}{\kappa}s^{-\kappa}$ where $\kappa=\lambda-1$.

In practice, function $P(s)$ is truncated by the minimum measureable slip, or slip resolution $s_{\min}>0$, and by the maximum slip size $s_{\max}<\infty$ measured during the total straining time. Since the avalanches are emitted with frequency $f$ [avalanches/s] under the assumption that the emission probability ascribes to the power-law relation $P\left( s \right)\propto s^{-\lambda}$, we consequently write that

$$f\left( s \right)=B s^{-\lambda} , (A5)$$

where the normalization constant $B$ is readily determined by enforcing $f\left( s_{\text{max}} \right)t=1$ for the largest avalanche emitted over time $t$. This avalanche size is then given by

$$s_{\text{max}}=\left( Bt \right)^{1/\lambda}. (A6)$$

The emission probability $P$ for avalanches, or slip events, emitted over a fixed bin width of size $\Delta s_{b}$ is calculated as $P\left( \bar{s} \right)=n/N$, where $n$ is the number of intermittencies in the bin width $\Delta s_{b}$, $N$ is the total number of slip events in the distribution and $\bar{s}$ is a mean slip value within the bin. A major drawback in this procedure is that the $\Delta s_{b}$ level appropriately binning large avalanche sizes is utterly excessive when compared to that required when binning small avalanches or slip events. Since function $C\left( s \right)$ can be conveniently obtained without any particular binning strategy, $P\left( s \right)$ is readily found through the derivative $dC\left( s \right)/ds$ (see Eq. (A4)). As $C\left( s \right)\propto s^{-\kappa}$, exponent $\kappa$ is then given by the slope of the log $(C)-$log $(s)$ plot, where$\lambda=\kappa+1$.

A discrete representation of function $C(s)$ is obtained by ranking the measured avalanches in ascending order $i=1, 2, \ldots,N$; so that ${i=1 \mathrm{for} s}_{\min}$ and $i=N \mathrm{for}s_{\text{max}}$. We then compute

$$C\left( s \right)=\sum_{i=1, \ldots,N; s_{i}\geq s} \frac{1}{N}=\frac{N_{\text{s}}}{N} , (A7)$$

where $N_{\text{s}}$ is number of slip events whose size is greater or equal than $s$. An advantage of taking $s_{i}\geq s$ rather than $s_{i}>s$ in the above summation is that $C\left( s_{\max} \right)=(1/N) > 0$, which implies that there shall always be a finite emission probability for a prospective avalanche carrying $s>s_{\max}$.

In the main text, we show that the $C\left( s \right)$ distributions exhibit two regimes that are sensibly fitted through the general formulation given in Eq. (3) from the main text, as prescribed by the cut-off slip size $s_{c}$. The power law exponent $\kappa$ is determined within the linear segment of the distribution (i.e., $s<$ $s_{c}$) so that the associated $\lambda=\kappa+1$.

An interesting property from the $C(s)$ distributions is that they scale with the minimum measurable slip $s_{\min}$ (or slip resolution) which yields $C\left( s_{\min} \right)=$ 1 (i.e., $P\left( s_{\min} \right)=0$). The shape of the function $C(s)$ is not therefore affected when the $s_{\min}$ value is changed, so that the cut-off slip $s_{c}$is preserved. This is visually illustrated in Fig. A3, where the selection of different $s_{\min}$ values shifts the $C(s)$ distributions along the vertical log $C\left( s \right)$-axis.

This scaling property for $C(s)$ is demonstrated next. First, we assume a set of avalanching event sizes $\{s_{1},s_{2},\ldots,s_{N}\}$ with empirical cumulative distribution function defined as

$$D\left( x \right)=\frac{1}{N}\sum_{i=1}^{N} H(x-s_{i}), (A8)$$

where $N$ is the total number of avalanche events and $H$ is the step function: $H\left( t \right)=1$ for $t\geq0$ and $H\left( t \right)=0$ for $t<0$. The associated complementary cumulative distribution function $D(x)=1-C(x)$ is then given by

$$C\left( x \right)=\frac{1}{N}\sum_{i=1}^{N} \bar{H}(x-s_{i}), (A9)$$

where the complementary step function is $\bar{H}\left( t \right)=1-H\left( t \right).$

1. (b)

*Fig. A3: Slip distributions obtained for different values of the slip resolution* $s_{min}$*. (a) Synthetic power-law distribution constructed for* $\kappa=0.5$ *with a total number of slip events* $N=100$*. The curve homogeneously shifts in the vertical* $C(s)$ *axis as the* $s_{\min}$ *level is gradually increased, where the total number of avalanches is then reduced from 100 to 10. Note that the overall shape of the* $C(s)$ *distribution remains invariant to the selected* $s_{\min}$*. (b) Avalanche distribution for FCC Ni (DDD simulations) again computed for different levels of the slip resolution* $s_{\min}$*. The figure visually illustrates the invariance of function* $C(s)$ *when the total number of avalanches decrease from 186 to 38 while* $s_{\min}$ *increases from* $\approx$ *0.1*$b$ *to 1*$b$*.*

Now consider the case where $m$ avalanching events lie beyond the experimental resolution $s_{\text{min}}$, so that the slip values $\{s_{1},\ldots,s_{\min}\}$ remain undetected. The new $C(s)$ distribution shall thus only contain $(N-m)$slip events. For the sake of simplicity and without loss of generality, we assume that the slip events are ranked in ascending order. The new complementary distribution function $\hat{C}\left( x \right)$is then given by

$$\hat{C}\left( x \right)=\frac{1}{N-m}\sum_{i=m+1}^{N} \bar{H}(x-s_{i}). (A10)$$

By comparing $C\left( x \right)$ and  $\hat{C}(x)$, one can write

$$\hat{C}\left( x \right)=\frac{N}{N-m}C\left( x \right) \mathrm{for}x>s_{\min} , (A11)$$

so that

$$\log\hat{C}\left( x \right)=\log N/{(N-m)}+\log C(x). (A12)$$

It is therefore concluded that when $s_{\min}$ is varied, the logarithmic representation of$C(s)$ is simply shifted by the magnitude $\log N/{(N-m)}$. It is noted that the above analysis applies to any functional form for $C(s)$, whose shape is therefore invariant to the selected $s_{\min}$ level. Moreover, this result implies that a single $C(s)$ master distribution can be constructed by conveniently shifting individual distributions from different experiments or simulations to various $s_{\min}$ levels along the vertical $C(s)$-axis (see Section 5).

Finally, it is noted that we use the KS test and the standard Bootstrapping method to establish whether a measured $C\left( s \right)$ distribution would exhibit a power-law regime for $s<s_{c}$, as well as in the finding of the statistical scatter of the associated $\kappa$ exponent. First, the standard Bootstrapping method is employed to construct 1000 resamples of the measured $C(s)$ curve, which are fitted to a power-law function for $s<s_{c}$ using the non-linear least squares method. This renders the $\kappa$ exponent of the measured distribution and the standard deviation of this $\kappa$ value. The KS test is then utilized to provide statistical support in that the measured $C\left( s \right)$ curve is indeed a power-law for $s<s_{c}$. This test uses a synthetically generated set of 5000 power-laws along with the tested $C\left( s \right)$ curve, which are all truncated at the same critical slip magnitude $s_{c}$ (i.e., we assume the power-laws are cut-off at $s_{\max}=s_{c}$ so that $C\left( s_{c} \right)\to0$). Note that since the truncation affects on the shape of the measured $C(s)$ distribution in the same manner as it affects the shape of the synthetically generated power-laws, the KS test remains statistically meaningful in the comparison of the distributions. In our analysis, the KS test is performed with a 5% significance level.

***4. Temporal analyses of avalanche emissions***

Further statistical analyses are currently performed by recourse to the avalanche emission frequency distribution function, defined as

$\nu\left( s \right)\equiv\frac{N_{s}}{t_{T}}$,$(A13)$

where $t_{T}$ is the total straining time. Since the total number of emitted avalanches is

$n_{T}=\frac{n\left( s_{\min} \right)}{C\left( s_{\min} \right)}$,$(A14)$

it then follows that

$\nu(s_{\min})={(n}_{T}/t_{T}) \times C(s_{\min}) , (A15)$

indicating that $\nu(s)$ is a power law function sharing the same geometrical features as function $C\left( s \right)$. Function $\nu(s)$, however, is a true physical quantity measured in avalanches per unit time, which is not influenced by the slip resolution. This can be readily seen since for $s>s_{\min}$, both $N_{s}$ and $t_{T}$ remain constant irrespective of the $s_{\min}$ level.

***5. On the finding of master*** $\boldsymbol{C(s)}$ ***distributions***

Following Fig. A3, it becomes evident that a single, master $C\left( s \right)$ distribution can be obtained by conveniently referring the data sets from individual experiments and simulations to the same $s_{\min}$ value, where $C\left( s_{\min} \right)=1$ is then enforced for the totality of the distributions. This is fulfilled by shifting each individual distribution along the vertical, logarithmic $C\left( s \right)$-axis, where the critical slip $s_{c}$ remains necessarily constant (Section 3). In contrast to the $C\left( s \right)$ distributions, the measured avalanche emission frequency function $\nu\left( s \right)$ has true physical meaning (i.e., number of avalanches emitted per unit time) and cannot be arbitrarily shifted.

The master $C(s)$ representation for *bulk-like microcrystal plasticity* in Fig. 2 from the main text is obtained by overlapping (i.e., shifting along the vertical ln$C\left( s \right)$-axis) the individual distributions measured with the Cu micropillars compressed along the hard [111] orientation (where the slip resolution $s_{\min}$ is of $\approx$ 1$b$ for $D=2$ μm); the MD simulations with Al cells performed under periodic boundary conditions at extreme dislocation densities (where the slip resolution $s_{\min}$ $\approx0.1b$); and the DDD simulations of [100]-oriented Ni micropillars containing a large dislocation density (where the slip resolution $s_{\min}$ $\approx$ $0.08b$). The $C(s)$ curve in Fig. 2 from the main text concerning large micropillars also deforming through *bulk-like plasticity* at larger $L_{c}$ is obtained from the Al microcrystals with $D=8$ μm (where the slip resolution $s_{\min}$ $\approx0.4b).$ The master $C(s)$ curve representing avalanches that propagate under *confined plasticity*, also shown in Fig. 2 from the main text, is obtained by overlapping (shifting) the individual distributions from the Cu micropillars which exhibited marked slip localization and markedly serrated stress-strain curves (where $s_{\min}$ $\approx2b)$. It is finally noted that in the case of the $C(s)$ distributions obtained from RT to 400 °C in FCC Cu micropillars whose diameters $D range from 0.6$ μm to $3.5$ μm in Fig. 3 from the main text, it is unnecessary to perform any shifting as all datasets share a common origin ($s_{\min}\approx4b$).

A similar scheme is adopted when plotting the distributions for the BCC microcrystals, where the slip resolutions are of $s_{\min}\approx$ 5*b* for the W and of $s_{\min}\approx$ 2*b* for the Ta micropillars (Fig. 5 in the main text). The difference in $s_{\min}$ between these microcrystals is due to the different elastic moduli $E$, while the resolution in the measurement of the stress fluctuations ($\Delta\sigma$) remains roughly constant (Eq. (A2)).

Figure A4 finally allows the comparison of the presently used slip distribution function $C(s)\propto s^{-\kappa}\exp\left( -s/s_{c} \right)$ with the function $P(s)\propto s^{-\kappa-1}\exp\left( -\left( s/s_{c} \right)^{2} \right)$ advocated in [4]. The analysis shows that the assumed exponent of 2 for the ratio $s/s_{c}$ does not fit the current distributions of the individual dislocation avalanche events. Along these lines, similar numeral fits as those from the currently used $C(s)$ function are obtained when the above exponent ranges from 0.8 to 1 (cf. Fig. 2 in the main text). The parameter $s_{c}$ then becomes consistent with a cut-off slip magnitude which effectively triggers exponential truncation of the slip distribution.

***6. Avalanche scaling in continuum plasticity***

As discussed in the main text, the coarse-grained strain hardening model for FCC crystals in Refs. [5$-$7] provides a mean value of the dislocation glide distance that is in agreement with the current magnitude of $L_{c}$ . This magnitude can be inferred from the DDD simulations for a plastic intermittency which carries the characteristic slip $s_{c}$ of the distribution. Invoking the simplified version of this model, it follows that

$L_{c}$=$K_{hkl}/\sqrt{\bar{a}\rho}$ *(A16)*

where $\sqrt{\bar{a}}\approx$0.35, $\rho$ is the total dislocation density in the crystal, and $K_{hkl}$ is a dimensionless constant that accounts for the expansion of the mobile dislocation segments against a dislocation forest, leading to the formation of the 4 types of dislocation junctions in FCC plasticity, in a crystal with the [$hkl$] orientation. It is found that $K_{111}=$ 7.29 and $K_{100}=$6.21, where the general formulation for the effective $K_{hkl}$ in any arbitrary oriented crystal is given in [6].

The effective dislocation density $\rho$ is then given by

$\tau_{\circ}=\mu b\sqrt{a\rho}$ *(A17)*

where $\tau_{\circ}$ is the shear stress level rendering plastic deformation in the active slip systems of the micropillars.

According to the dislocation storage-recovery model, it then follows that

$\frac{d\rho}{d\gamma}=\frac{1}{b}\left( \frac{1}{L}-y\rho\right)$ *(A18)*

where the parameter $y$ accounts for the mutual dislocation annihilation attained through cross-slip.

***7. Introduction of dislocation networks in the MD simulations***

The stress$-$strain curves from the MD simulations were obtained with cuboidal cells containing a pre-existing dislocation network that was introduced by bringing one of the surfaces into contact against a spherical indenter [8]. The simulation cell size was 70 $\times$ 70 $\times$ 40 nm comprising $\approx$11 million atoms for Al and $\approx$10 million atoms for Ta, modelled through the embedded-atom method (EAM) potentials in Refs. [49,50] from the main text. Upon application of periodic boundary conditions to the simulation cells, the resulting stress$-$strain curves were taken to represent bulk material responses as described in the following section. All simulations were carried out with the LAMMPS code [9].


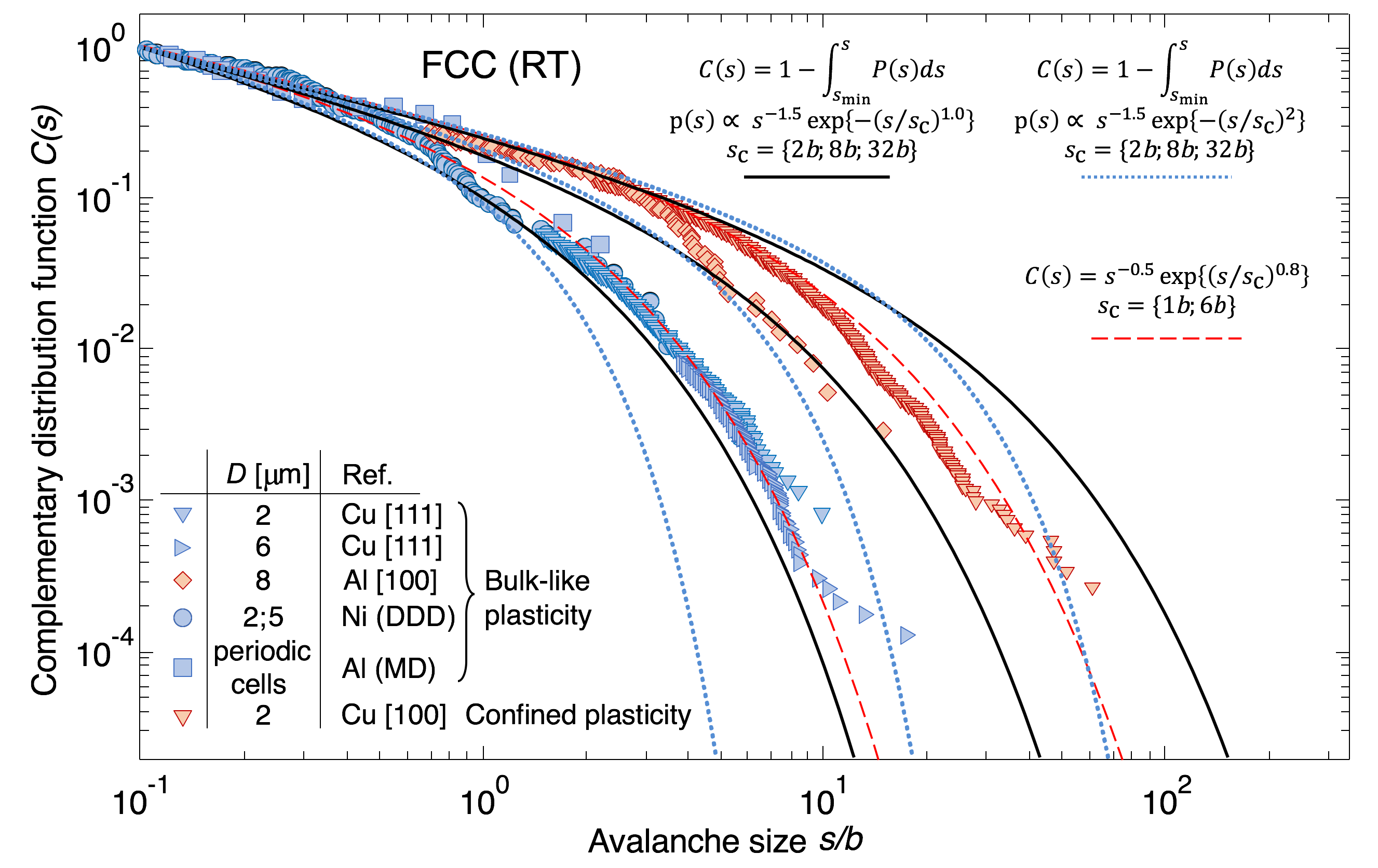


*Fig. A4: Fitting of the probability density function* $P\left( s \right) \sim s^{-1.5}\exp\left( -\left\{ \frac{s}{s_{c}} \right\}^{n} \right)$ *to the individual avalanche events from the* $C\left( s \right)$ *distributions in Fig. 2 of the main text. Note that* $n=1$ *rather than the value of* $n=2$ *advocated in Ref. [4] provides a better description to the actual cut-off of the individual avalanche events. Moreover, note that the above* $P\left( s \right)$ *function with*$n=1$ *provides nearly identical numerical fits as the function* $C\left( s \right) \sim s^{-1.5}\exp\left( -\left\{ \frac{s}{s_{c}} \right\} \right)$ *employed in the main text (where the ratio* $s/s_{c}$ *goes to the power of one).*

The nanocontact loading was imposed through a repulsive potential reproducing a spherical tip with a diameter of 48 nm, which was forced to penetrate against the (111)-oriented Al and (011)-oriented Ta simulation cells to a maximum contact radius of $\approx$ 18 nm at 4 m/s under the NVT canonical ensemble. Periodic boundary conditions were imposed to the four lateral surfaces of the domains while suppressing atomic motion in the bottom surface. The near surface indentation-induced defect structure was then eliminated by removing a $\approx$7 nm-thick layer of material. Energy minimization was subsequently applied by recourse to the steepest descent relaxation algorithm, leading to a final dislocation density, $\rho$, of ${10}^{16}$ $m^{-2}$. To facilitate dislocation network development and dissemination across the simulation cells, a uniaxial tensile strain $\varepsilon\approx3\%$ was preliminary applied at 0.8 m/s along the [110] and [100] orientations of the Al and Ta MD boxes, respectively. The final stress$-$strain curves were obtained by subjecting the simulation cells to a compressive strain $\varepsilon\approx$3% at RT in Al and at RT and 630°C in Ta under an imposed displacement rate of 0.1 m/s (see Section 1). All simulations were performed under the NPT canonical ensemble using Nosé-Hoover chains and timestep of 1 fs. A full round of simulations was further performed with cuboidal cells with four lateral free surfaces to ascertain salient features of surface slip trace patterning.

Attention was given to assess the strengthening properties of the currently introduced dislocation networks as compared to those arising at much smaller levels of dislocation density in macroscopic material samples. This was carried-out by invoking Taylor hardening model, $\tau=\alpha Gb\sqrt{\rho}$, where $\alpha$ is the dislocation interaction coefficient measuring the strength of the dislocation junctions, $\tau$ is the resolved shear stress in the active slip systems in which the avalanches propagate and $G$ is the shear modulus. The value of $\alpha$ was found to be 0.35 at the onset of yielding in the simulated stress-strain curve, in excellent agreement with the reported values in pure FCC crystals containing less entangled networks where $\rho\approx$ 10^8^ to 10^12^ m^-2^ [10]. The strengthening properties of the injected networks thus naturally scale with dislocation density irrespective of the specificities of dislocation clustering or network arrangement in the simulation cells.

**8*. Representative volume elements in the MD simulations***

In all simulations performed under periodic boundary conditions, the material sample is conceivably comprised of a connected 3D array of MD domains. The computational cell thus acts as a representative material volume element ensuring that (i) the active dislocations exit and reentry the MD cell exit at different locations, thus interacting against different arrangements of immobile obstacles so as to become arrested upon a single excursion –this prevents recursive avalanching within one volume element, (ii) the criticality of the embodied (bulk) sample remains size-independent as a plastic slip is simultaneously reproduced in all connected representative elements; and (iii) the largest dislocation avalanche sweeping across the computational domain produces the largest slip magnitude when it propagates across the entire microcrystal of characteristic size $D$ (see Eq. 2 in the main text).

***References***

1. P.S. Phani and W.C. Oliver, Ultra high strain rate nanoindentation testing, *Materials* 10 663 (2019).
2. D.M. Dimiduk, M.D. Uchic, T.A. Parthasarathy, Size-affected single-slip behavior of pure nickel microcrystals, *Acta Materialia* 53 4065–4077 (2005).
3. S. Peng, Y. Wei, Z. Jin, W. Yang, Supersonic screw dislocations gliding at the shear wave speed, *Phys. Rev. Lett*. 122, 045501 (2019).
4. F.F. Csikor, C. Motz, D. Weygand, M. Zaiser, S. Zapperi, Dislocation avalanches, strain bursts, and the problem of plastic forming at the micrometer scale, *Science* **318**, 251-254 (2007).
5. B. Devincre, T. Hoc and L. Kubin, Dislocation mean free paths and strain hardening of crystals, *Science* **320**, 1745-1748 (2008).
6. L. Kubin, B. Devincre, T. Hoc, Modeling of dislocation storage rates and mean free paths in face-centered cubic crystals, *Acta Mater*. **56**, 6040–6049 (2008).
7. L. Kubin, T. Hoc, B. Devincre, Dynamic recovery and its orientation dependence in face-centered cubic crystals, *Acta Mater*. **57**, 2567–2575 (2009).
8. J. Alcalá, J. Ocenasek, K. Nowag, D.E. de los Ojos, R. Ghisleny, J. Michler, Strain hardening and dislocation avalanches in micrometer-sized dimensions, *Acta Mater.* **91** 255-266 (2015).
9. S. Plimpton, Fast Parallel Algorithms for Short-Range Molecular Dynamics, J Comp Phys, 1–19 (1995).
10. Basinski, S.J. and Basinski, Z.S. Plastic deformation and work hardening. In Dislocations in Solids 4, (North-Holland Publishing Company, 1979).

Supporting Movie S1: MD simulation of an avalanche event in FCC Al at RT compressed at 0.1 m/s.

Supporting Movie S2: MD simulation of an avalanche event in BCC Ta at RT compressed at 0.1 m/s.

Supporting Movie S3: MD simulation of an avalanche event in BCC Ta at 630 $℃$ compressed at 0.1 m/s.
